# The SZT2 Interactome Unravels New Functions of the KICSTOR Complex

**DOI:** 10.3390/cells10102711

**Published:** 2021-10-09

**Authors:** Cecilia Cattelani, Dominik Lesiak, Gudrun Liebscher, Isabel I. Singer, Taras Stasyk, Moritz H. Wallnöfer, Alexander M. Heberle, Corrado Corti, Michael W. Hess, Kristian Pfaller, Marcel Kwiatkowski, Peter P. Pramstaller, Andrew A. Hicks, Kathrin Thedieck, Thomas Müller, Lukas A. Huber, Mariana Eca Guimaraes de Araujo

**Affiliations:** 1 Institute of Cell Biology, Biocenter, Medical University of Innsbruck, 6020 Innsbruck, Austria; Cecilia.Cattelani@i-med.ac.at (C.C.); Dominik.Lesiak@student.i-med.ac.at (D.L.); Gudrun.Liebscher@i-med.ac.at (G.L.); Isabel.Singer@i-med.ac.at (I.I.S.); Taras.Stasyk@i-med.ac.at (T.S.); moritz.wallnofer@student.i-med.ac.at (M.H.W.); Lukas.A.Huber@i-med.ac.at (L.A.H.); 2 Institute for Biomedicine, Eurac Research, Affiliated Institute of the University of Lübeck, 39100 Bolzano, Italy; Corrado.Corti@eurac.edu (C.C.); Peter.Pramstaller@eurac.edu (P.P.P.); Andrew.Hicks@eurac.edu (A.A.H.); 3 Institute of Biochemistry and Center for Molecular Biosciences Innsbruck, University of Innsbruck, 6020 Innsbruck, Austria; Alexander.Heberle@uibk.ac.at (A.M.H.); Marcel.Kwiatkowski@uibk.ac.at (M.K.); Kathrin.Thedieck@uibk.ac.at (K.T.); 4 Laboratory of Pediatrics, Section Systems Medicine of Metabolism and Signaling, University of Groningen, University Medical Center Groningen, 9700 RB Groningen, The Netherlands; 5 Institute of Histology and Embryology, Medical University of Innsbruck, 6020 Innsbruck, Austria; Michael.Hess@i-med.ac.at (M.W.H.); Kristian.Pfaller@i-med.ac.at (K.P.); 6 Department for Neuroscience, School of Medicine and Health Sciences, Carl von Ossietzky University Oldenburg, 26129 Oldenburg, Germany; 7 Department of Pediatrics I, Medical University of Innsbruck, 6020 Innsbruck, Austria; Thomas.Mueller@i-med.ac.at; 8 Austrian Drug Screening Institute, ADSI, 6020 Innsbruck, Austria

**Keywords:** KICSTOR, mTORC1, SZT2, epilepsy, neurogenesis, autophagy, ciliogenesis, neurodegeneration

## Abstract

Seizure threshold 2 (SZT2) is a component of the KICSTOR complex which, under catabolic conditions, functions as a negative regulator in the amino acid-sensing branch of mTORC1. Mutations in this gene cause a severe neurodevelopmental and epileptic encephalopathy whose main symptoms include epilepsy, intellectual disability, and macrocephaly. As SZT2 remains one of the least characterized regulators of mTORC1, in this work we performed a systematic interactome analysis under catabolic and anabolic conditions. Besides numerous mTORC1 and AMPK signaling components, we identified clusters of proteins related to autophagy, ciliogenesis regulation, neurogenesis, and neurodegenerative processes. Moreover, analysis of SZT2 ablated cells revealed increased mTORC1 signaling activation that could be reversed by Rapamycin or Torin treatments. Strikingly, SZT2 KO cells also exhibited higher levels of autophagic components, independent of the physiological conditions tested. These results are consistent with our interactome data, in which we detected an enriched pool of selective autophagy receptors/regulators. Moreover, preliminary analyses indicated that SZT2 alters ciliogenesis. Overall, the data presented form the basis to comprehensively investigate the physiological functions of SZT2 that could explain major molecular events in the pathophysiology of developmental and epileptic encephalopathy in patients with SZT2 mutations.

## 1. Introduction

Human SZT2 encodes a 3375 amino acid protein, highly conserved throughout evolution [1], with a unique orthologue found in most vertebrate species. It is predominantly expressed in the brain (parietal and frontal cortex, dorsal root ganglia) but can be found at lower levels in most other tissues [2]. SZT2 has been implicated in epileptogenesis in humans and mice [1], in resistance to oxidative stress, and more recently as a negative regulator of the mTORC1 signaling pathway [3,4]. In brief, SZT2 forms a complex named KICSTOR with KPTN, ITFG2, and C12orf66. This complex coordinates the sensing of amino acid depletion, together with GATOR1 and GATOR2, and inactivates mTORC1 under catabolic conditions by recruiting GATOR1 to the lysosome. In the absence of KICSTOR, mTORC1 is constitutively bound to the lysosomal membrane and activated regardless of nutrients availability [3,4].

From a structural perspective, SZT2 contains a PTS1 (peroxisomal targeting signal) and a predicted superoxide dismutase motif, for which no activity has yet been shown [2]. Despite these annotated domains, SZT2 did not colocalize with the peroxisomal marker PMP7026 by immunofluorescence [4]. Structure prediction tools failed to identify the presence of other domains, and it remains unclear which regions of SZT2 contribute towards KICSTOR formation. Importantly, deletion of residues 1026 to 1132 was shown to prevent binding to GATOR1, while the C-terminus of the protein was shown to contribute towards the regulation of mTORC1 signaling [4].

The mechanistic target of Rapamycin complex 1 (mTORC1) regulates the balance between biosynthetic and catabolic processes influencing not only cellular metabolism, but also growth, proliferation, and survival. Studies in different organisms have reported localization of mTORC1 to distinct subcellular compartments including peroxisomes [5], ER, Golgi [6,7], nucleus, cytoplasm [8,9,10], and late endosomes/lysosomes, the latter are probably the best studied intracellular localizations [11,12]. Lysosomal mTORC1 activation is triggered as a coordinated response to amino acid and glucose availability (through the Rag GTPases signaling pathway) [12,13,14,15], growth factors (through inhibition of the tuberous sclerosis protein complex (TSC) and activation of Rheb [16,17]), and cellular energy status (through AMPK phosphorylation of TSC2 and Raptor [18,19]). Importantly, simultaneous recognition of these different input signals ensures that the catalytic activity of mTORC1 is triggered only under fully anabolic conditions that allow cell growth.

The recruitment of Raptor, and thereby mTORC1, to lysosomes depends on the presence and activation of the Rag GTPases, hereafter Rags, on the organelle membrane [12,20,21]. The Rags form obligate heterodimers and, unlike other Ras-superfamily GTPases, they do not directly associate with the lysosomal membrane, but require the presence of a scaffolding complex named LAMTOR [11]. LAMTOR stands for late endosomal and lysosomal adaptor and mitogen activated protein kinase (MAPK) and mTORC activator. In addition to its role in mTORC1 activation, the complex also recruits growth factor dependent ERK signaling to lysosomes [22,23,24,25]. In the absence of amino acids or growth factors, the TSC complex is recruited to lysosomes where it prevents the activation of the small GTPase Rheb [26,27,28,29]. In contrast, the presence of amino acids allows the release of TSC, permitting the interaction between translocated mTORC1 and Rheb. This interaction is fundamental for the catalytic activation of the kinase and the phosphorylation of downstream targets [30,31]. Efforts by us and others led to the identification of SLC38A9, a lysosomal transceptor that, together with the vATPase, conveys inputs from the lysosomal luminal amino acid pool [32,33,34] in a so called inside-out sensing mechanism. Additionally, cytosolic amino acids, in particular arginine, leucine, and s-adenosylmethionine, are sensed by the recently identified complexes CASTOR1, Sestrin 2, and SAMTOR and the mediators GATOR1 and GATOR2 [35,36,37,38,39]. Importantly, the amino acid sensing branch converges on the Rags, thus strengthening the critical role of the heterodimer in mTORC1 activation.

The hyperactivation of the mTOR signaling pathway has been related to different epileptogenic conditions (mTORopathies) such as tuberous sclerosis (TSC, caused by TSC1 or TSC2 mutations), autosomal dominant nocturnal frontal lobe epilepsy (caused by DEPDC5 mutations), PIK3CA-related overgrowth spectrum (PROS), megalencephaly-polymicrogyria-polydactyly-hydrocephalus syndrome-2 (caused by AKT3 mutations), Smith–Kingsmore syndrome (caused by mTOR mutations), focal cortical dysplasia type II (caused by mTOR, TSC1, or TSC2 mutations), or familial focal epilepsy with variable foci (caused by DEPDC5, NPRL2, or NPRL3 mutations) [40,41].

Developmental and epileptic encephalopathy (DEE) is a group of rare, severe neurodevelopmental disorders characterized by the co-occurrence of epilepsy and intellectual disability (ID), in which there is additional developmental impairment independent of epileptic activity [42,43]. Recent advances in genomics technology have enabled the identification of new causal genes and variants for this disease. Nevertheless, even the most thorough exome-sequencing studies leave 60–65% of patients without a molecular diagnosis.

In 2009, a chemical mutagenesis screen in mice found that mutations in SZT2 conferred a higher susceptibility to seizures in a semi-dominant manner [1]. Since then, 17 independent reports have identified 28 patients with bi-allelic SZT2 mutations (in compound heterozygous or homozygous state), diagnosed with DEE. Their symptoms include developmental delay of varying severity, hypotonia, and dysmorphic features such as macrocephaly, often associated with corpus collosum abnormalities. Patients commonly suffer from recurrent epileptic seizures that begin at infancy and are frequently refractory to available therapies.

As SZT2 remains one of the least characterized regulators of mTORC1, we began to decipher its interactome under catabolic and anabolic conditions. This comprehensive overview of SZT2 interactors captured the complex biological networks the protein is involved in, and highlighted a number of key interaction nodes that might have a role in the etiology of DEE. Additional studies will be necessary to expand these findings and obtain more detailed mechanistic insight.

## 2. Materials and Methods

### 2.1. Cell Lines, Cell Culture, and Treatment of Cells

HEK293, HEK293LTV, and MDCK cells were cultured in Dulbecco’s modified Eagle Medium (DMEM) (D6429 Sigma-Aldrich Handels Gmbh, Vienna, Austria), supplemented with 10% (*vol/vol*) FBS (10270 ThermoFisher, Life Tecnologies Austria Zweigniederlassung, Vienna, Austria) and antibiotics (100 U/mL penicillin and 100 µg/mL streptomycin) (P0781 Sigma-Aldrich Handels Gmbh, Vienna, Austria). The HEK293 Flp-In™ T-Rex™ (R78007 ThermoFisher, Life Tecnologies Austria Zweigniederlassung, Vienna, Austria) were grown in DMEM (D6429 Sigma-Aldrich Handels Gmbh, Vienna, Austria), supplemented with 10% (*vol/vol*) FBS (10270 ThermoFisher, Life Tecnologies Austria Zweigniederlassung, Vienna, Austria), 100 U/mL penicillin and 100 µg/mL streptomycin (P0781 Sigma-Aldrich Handels Gmbh, Vienna, Austria), 100 µg/mL Zeocin (ant-zn-5b InvivoGen, Toulouse, France) and 15 µg/mL Blasticidin (ant-bl-5b InvivoGen, Toulouse, France). The HEK293 Flp-In™ T-Rex™ expressing SH tagged SZT2 WT were cultured in DMEM (D6429 Sigma-Aldrich Handels Gmbh, Vienna, Austria), supplemented with 10% (*vol/vol*) FBS (10270 ThermoFisher, Life Tecnologies Austria Zweigniederlassung, Vienna, Austria), 100 U/mL penicillin and 100 µg/mL streptomycin (P0781 Sigma-Aldrich Handels Gmbh, Vienna, Austria), 100 µg/mL Hygromycin (ant-hg-5 InvivoGen, Toulouse, France), and 15 µg/mL Blasticidin (ant-bl-5b InvivoGen, Toulouse, France). All cell lines were maintained in an incubator at 37 °C, 5% CO_2_, and 95% relative humidity, and routinely tested for the absence of mycoplasm.

To address the impact of SZT2 depletion in mTORC1 signalling (Western blotting and cell diameter analysis), cells were rinsed twice in PBS and starved of amino acids for 1 h in DMEM/Ham’s F-12 medium (D9811-01 US Biological Life Sciences, Salem, MA, USA) supplemented with 10% dialyzed FBS (26400044 ThermoFisher, Life Tecnologies Austria Zweigniederlassung, Vienna, Austria). For restimulation, cells were kept in DMEM/Ham’s F-12 medium (D9811-01 US Biological Life Sciences) supplemented with 10% dialyzed FBS (26400044 ThermoFisher), essential amino acids (11130036 ThermoFisher, Life Tecnologies Austria Zweigniederlassung, Vienna, Austria), non-essential amino acids (11140035, ThermoFisher, Life Tecnologies Austria Zweigniederlassung, Vienna, Austria), and glutamine (25030-024 ThermoFisher, Life Tecnologies Austria Zweigniederlassung, Vienna, Austria), for 20 min. When applicable, Torin (Cay10997-10 Cayman Chemical, Ann Arbor, MI, USA) and Rapamycin (R-5000 Biozol, Leipziger, Germany) were added at 330 nM and 40 nM, respectively.

For the interactome analysis and the cell diameter experiments, cells were rinsed twice in PBS and subsequently starved for amino acids and growth factors for the indicated time points using DMEM/Ham’s F-12 medium (D9811-01 US Biological Life Sciences, VWR International Gmbh, Vienna, Austria). Cells were subsequently restimulated in DMEM (D6429 Sigma-Aldrich Handels Gmbh, Vienna, Austria), supplemented with 10% (*vol/vol*) FBS (10270 ThermoFisher, Life Tecnologies Austria Zweigniederlassung, Vienna, Austria) for the indicated time points. When specified, cells were treated with 40µM Chloroquine (C6628 Sigma-Aldrich Handels Gmbh, Vienna, Austria) and 40 nM Bafilomycin A (BML-CM110-0100 Enzo Life Sciences, New York, NY, USA) for 6 h.

Finally, for the ciliogenesis experiments, cells were rinsed twice in PBS and subsequently starved for serum for 72 h using DMEM medium (D6429 Sigma-Aldrich Handels Gmbh, Vienna, Austria) without supplements.

### 2.2. Generation of HEK293 Flp-In™ T-Rex™ Cell Line Inducibly Expressing SH.SZT2WT

The pRK5-HA-SZT2 was purchased from Addgene Europe, Teddington, UK (No. 87035, provided by David Sabatini) [3]. The plasmid was digested with EcoRI and NotI enzymes, and the HA-SZT2 sequence was subcloned into the respective sites of pCDNA3.1 neo (V79020 ThermoFisher, Life Tecnologies Austria Zweigniederlassung, Vienna, Austria). Gateway recognition sites were then inserted into the plasmid flanking the HA-SZT2 coding sequence: the attL1 site was inserted between the EcoRI and AflII restriction enzymes cutting sites, the attL2 site between the XbaI and NotI sites. Recombination via Gateway LR Clonase II enzyme (11791020 ThermoFisher, Life Tecnologies Austria Zweigniederlassung, Vienna, Austria) was then performed between the generated entry vector and pcDNA™FRT⁄TO/SH/GW [34], giving rise to an expression construct containing the SZT2 coding sequence with a STREP-HA tag at the N terminus. All generated constructs were sequence verified.

HEK293 Flp-In™ T-Rex™ (R78007 ThermoFisher, Life Tecnologies Austria Zweigniederlassung, Vienna, Austria) were grown to 60% confluence and transfected with the generated expression plasmid and a pOG44 vector, containing the Flp Recombinase (V600520 Thermofisher, Life Tecnologies Austria Zweigniederlassung, Vienna, Austria). Transfection was performed using Lipofectamine LTX (15338100 ThermoFisher, Life Tecnologies Austria Zweigniederlassung, Vienna, Austria) and Opti-MEM Reduced Serum medium (31985070 ThermoFisher, Life Tecnologies Austria Zweigniederlassung, Vienna, Austria). Selection was then carried out for 3 weeks in DMEM (D6429 Sigma-Aldrich Handels Gmbh, Vienna, Austria), supplemented with 10% (*vol/vol*) FBS (10270 ThermoFisher, Life Tecnologies Austria Zweigniederlassung, Vienna, Austria), 100 U/mL penicillin and 100 µg/mL streptomycin (P0781 Sigma-Aldrich Handels Gmbh, Vienna, Austria), 100 µg/mL Hygromycin (ant-hg-5 InvivoGen, Toulouse, France) and 15 µg/mL Blasticidin (ant-bl-5b InvivoGen, Toulouse, France). The cell line was subsequently expanded and consolidated. In order to titrate SH.SZT2WT expression, the cells were induced in increasing concentrations of Tetracyclin (T7660-5G Sigma-Aldrich Handels Gmbh, Vienna, Austria) 24 h prior to harvesting. From this analysis, we selected 40 ng/mL as the Tetracycline concentration for the purification experiments.

### 2.3. Affinity Purification and Sample Preparation for Mass Spectrometry

The HEK293 Flp-In™ T-Rex™ cell lines inducibly expressing either SH.GFP or SH.SZT2WT were expanded to 5 × 15 cm cell culture dishes (145 cm²/dish) per biological replicate. Cells were induced with 40 ng/mL Tetracycline (T7660-5G Sigma-Aldrich Handels Gmbh, Vienna, Austria) in cultivation medium without Hygromycin or Blasticidin, 24 h before harvesting. Cells were then subjected to starvation and restimulation with amino acids and FBS as described above (Section 2.1). Prior to harvesting, the cultivation medium was removed, and the cells were washed twice in ice cold PBS. Cells were detached with the help of a cell scraper and the content of the 5 dishes pooled into a single tube. Upon centrifugation, the obtained pellets were snap-frozen in liquid nitrogen and stored at −80 °C.

The SH-affinity purification was performed exclusively on ice. The cell pellets were thawed and resuspended in lysis buffer (50 mM HEPES pH 8.0, 150 mM NaCl, 5 mM EDTA pH 8.0, 0.5% NP-40, 50 mM NaF, 10 µg/mL Leupeptin, 0.4 mM Pefablock, 1 µg/mL Pepstatin, 0.5 mM PMSF, 1.5 µg/mL Avidin, 1 mM Na3VO4). After 30 min incubation at 4 °C, the cell lysates were centrifuged for 15 min at 13,000× *g*, 4 °C. Supernatants were collected into fresh tubes and the centrifugation was repeated. Upon clearance, a 50 µL aliquot from each supernatant was collected for a posteriori analysis. The protein concentration of the remaining lysates was normalized prior to the subsequent steps. Strep-Tactin^®^ Sepharose^®^ beads, 600 µL per sample (2-1201-002 IBA Lifesciences, Göttingen, Germany) were transferred to fresh tubes, washed twice, and equilibrated in 50 mM HEPES pH 8.0, 150 mM NaCl, 5 mM EDTA pH 8.0, 0.5% NP-40, 50 mM NaF. The washing steps were intercalated with 3 min centrifugations at max. 300× *g*, 4 °C. The normalized lysates were then added to the beads and the suspensions incubated for 1 h, rotating at 20 RPM and 4 °C. After incubation, the beads were centrifuged at max. 300× *g*, 4 °C. A 50 µL aliquot of each supernatant was collected as “flow through” for later analysis, and the rest of the supernatant was aspirated with a sterile 25 G × 1 ½˝ needle. The needle was changed between samples to avoid cross-contamination. The beads were then washed once in washing buffer (50 mM HEPES pH 8.0, 150 mM NaCl, 5 mM EDTA pH 8.0, 0.5% NP-40, 50 mM NaF, 10 µg/mL Leupeptin, 0.4 mM Pefablock, 1 µg/mL Pepstatin) and transferred into one pre-equilibrated ‘Bio-Spin Disposable Chromatography Column’ (7326008 Biorad, Hercules, CA, USA) per sample. Then, the columns were washed 6 times with washing buffer followed by 2 times with 50 mM HEPES pH 8.0, 150 mM NaCl, 5 mM EDTA pH 8.0, and 0.1% NP-40. Finally, the proteins bound to the beads were eluted in 300 µL of 2.5 mM D-Biotin, 55 mM HEPES pH 8.0, 135 mM NaCl, 4.5 mM EDTA pH 8.0, and 0.1% NP-40 into 1.5 mL ‘Protein LoBind^®^ Tubes’ (EP0030122216 Sigma-Aldrich Handels Gmbh, Vienna, Austria). The elution step was repeated 3 times. Two 50 µL aliquots of the eluate were kept aside for quality control purposes. The remaining eluate samples were frozen in liquid nitrogen and stored at −20 °C until further processing. To address if elution was complete, the beads were then incubated in 300 µL of SDS sample buffer without DTT (272 mM Tris-HCl pH 6.8, 70% glycerol, 8% SDS, 0.02% bromophenol blue). The solution was collected into 1.5 mL tubes and the process was repeated two more times. For Western blot analysis, 25 µL of 5× sample buffer (250 mM Tris-HCl pH 6.8, 150 mM Glycerol, 0.05% Bromphenol Blue, and 10% SDS, 500 mM DTE) and 50 µL of 2 M Urea lysis buffer (2 M Urea, 50 mM Tris-HCl pH 8.0, 1% SDS, 1 mM EDTA pH 8.0, and 10 mM NaF) were added to each of the quality control aliquots. These were denatured by heating at 60 °C for 10 min.

Affinity purified SZT2 interacting proteins were precipitated with the TCA-DOC method [44] and reconstituted in SDS-containing sample buffer. Cysteines were reduced by incubating the samples in the presence of 20 mM DTT for 5 min at 95 °C and alkylated with 55 mM iodoacetamide as described previously [45]. Six independent biological replicates for each condition, plus 6 corresponding to GFP control samples, were loaded on 10% Tris-HCl polyacrylamide gels and run for 30 min until the dye front penetrated 2 cm into the resolving gel. Coomassie stained gels were sliced into 3 to 5 fractions as shown in Figure S1A, further chopped to approximately 1 mm^3^ pieces and in-gel digested with trypsin followed by mass spectrometry.

### 2.4. LC-MS/MS Analysis

For liquid chromatography tandem mass spectrometry (LC-MS/MS) analysis, the dried tryptic peptides were dissolved in 20 µL 0.1% FA. The samples were injected into a nano-ultra pressure liquid chromatography system (Dionex UltiMate 3000 RSLCnano pro flow, Thermo Scientific, Bremen, Germany) coupled via an electrospray ionization (ESI) source to an Orbitrap QExactive HFX (Thermo Scientific, Bremen, Germany). The samples were loaded (5 µL/min) on a trapping column (nanoE MZ Sym C18, 5 μm, 180 µm × 20 mm, Waters, Germany, buffer A: 0.1% FA in HPLC-H_2_O; buffer B: 80% ACN, 0.1% FA in HPLC-H_2_O) with 5% buffer B. After sample loading, the trapping column was washed for 5 min with 5% buffer B (15 μL/min) and the peptides were eluted (250 nL/min) onto the separation column (nanoE MZ PST CSH, 130 A, C18 1.7 μ, 75 μm × 250 mm, Waters, Germany) and separated with a gradient of 5−37.5% B in 30 min. The spray was generated from a steel emitter (Fisher Scientific, Dreieich, Germany) at a capillary voltage of 1850 V. MS/MS measurements were carried out in data dependent acquisition mode (DDA) using a normalized HCD collision energy of 28% and a loop count of 15. MS scan was performed over an *m/z* range from 350–1600, with a resolution of 60,000 at *m/z* 200 (maximum injection time = 50 ms, AGC target = 3 × 10^6^). MS/MS spectra were recorded over a *m/z* range of 120–2000 *m/z* with a resolution of 7500 at *m/z* 200 (maximum injection time = 50 ms, maximum AGC target = 1 × 10^5^, intensity threshold: 8 × 10^3^), a quadrupole isolation width of 0.8 Da and an exclusion time of 60 s.

### 2.5. Data Analysis

LC-MS/MS raw files were analyzed with ProteomeDiscoverer 2.4 (Thermo Fisher Scientific, San Jose, CA, USA). For peptide and protein identification, SequesHT was used to search against a human reference database (SwissProt, 20,369 entries) and a contaminant database (116 entries). The following parameters were used for the database search: mass tolerance MS1: 6 ppm, mass tolerance MS2: 0.02 Da, fixed modification: carbamidomethylation (Cystein), variable modification: Oxidation (Methionine), and variable modification at protein N-terminus: Acetylation, Methionine loss, and Methionine loss + Acetylation. Percolator was used for FDR calculation. For feature detection, Minora Feature Detection was used with default settings. For label free quantification, the Precursor Ions Quantifier was used with the following parameters: Peptides to use: unique peptides, Precursor Abundance Based On: Area, Minimum Replicate Features: 50%, Normalization Mode: Total Peptide Amount, Protein Abundance Calculation: Summed Abundances, Top N: 3, and Hypothesis testing: *t*-test (Background Based). Adjusted *p*-values were calculated using Benjamini–Hochberg correction.

Principal component analysis was performed using Genesis (v.1.8.1, Institute for Genomics and Bioinformatics, Graz University of Technology, Graz, Austria) [46]. For each identified protein the abundancies in all measured fractions of each replicate were summed and used as a value for calculating the principal components. The number of principal components was selected based on the percentage of variability they describe, which should be above 60%. The resulting clusters were depicted in a 2D-Component1 versus Component2 plot. The abundancy ratio of proteins binding to SZT2 over the GFP control was plotted against their respective *p*-value in volcano plots.

Pathway analysis was performed using Cytoscape (v. 3.8.2., Cytoscape Consortium, San Diego, CA, USA) and the plugin CLUEGO (v. 2.5.8, Laboratory of Integrative Cancer Immunology, Graz University of Technology, Graz, Austria) [47] based on the pathway databases Kyoto Encyclopedia of Genes and Genomes (KEGG pathways updated 6 July 2021) and Wikipathways (updated 5 July 2021). Terms were only included if their *p*-values were below 0.05 (two-sided hypergeometric test), they contained a least 7 of the identified proteins and these proteins made up at least 10% of all proteins associated with this term. The map nodes were depicted based on the number of genes and the significance value. For analysis of proteins preferentially binding to SZT2 upon starvation or upon stimulation, the protein nodes were colored based on their abundancy ratio.

### 2.6. Generation of KO Cell Lines

The sgRNA for SZT2 KO targeting exon 1 (sequence: CTCCGGCTCCGGGCGCTCCG) has been reported elsewhere [4]. We subcloned the sgRNA into pLentiCRISPRv2 LoxPv1 [48] via digestion with BsmBI restriction enzyme (FD0454 ThermoFisher, Life Tecnologies Austria Zweigniederlassung, Vienna, Austria). The construct was sequence verified prior to usage. HEK293 cells and HEK293 Flp-In™ T-Rex™ were grown to 70% confluence and transfected with the generated constructs using Lipofectamine 3000 (L3000015 ThermoFisher, Life Tecnologies Austria Zweigniederlassung, Vienna, Austria) and Opti-MEM Reduced Serum medium (31985070 ThermoFisher, Life Tecnologies Austria Zweigniederlassung, Vienna, Austria). 72 h later, single cell clones were isolated via a serial dilution method and expanded in 24-well plates. Once confluent, half of the culture was pelleted for genomic DNA extraction and the other half was replated to maintain the clone. Genomic DNA was purified using DirectPCR Lysis reagent (Mouse Tail) (102-T Viagen Biotech, Los Angeles, CA, USA) with Proteinase K (P8044 Sigma-Aldrich Handels Gmbh, Vienna, Austria) for 3 h at 55 °C. Genomic DNA was then utilized in the screening PCR employing Taq Polymerase (homemade) and the primer strategy summarized in Table 1.

Positive clones were later genotyped using the primer strategy annotated in Table 1, by cloning the fragment corresponding to the SZT2 N-terminal and upstream region into pCR™-Blunt II-TOPO vector (451245 ThermoFisher, Life Tecnologies Austria Zweigniederlassung, Vienna, Austria). A minimum of 10 colonies per cell clone were sequence verified to assess the specific editing on that locus and assure the absence of endogenous untargeted SZT2 alleles.

To generate the SZT2 KO MDCK cell line, HEK293LTV cells were used for lentiviral production. These were cotransfected with the pLentiCRISPRv2-LoxPv1 plasmid containing the sgRNA against SZT2 described above, together with pVSV-G (631530 Clontech, Takara Bio Europe SAS, Saint-Germain-en-Laye, France) and psPAX2. The supernatant containing the generated viral particles was used to infect MDCK cells in suspension. Three days later, the cells were selected with 4 µg/mL puromycin (P8833-25MG Sigma-Aldrich Handels Gmbh, Vienna, Austria). Genomic DNA of the bulk population was purified as described above and utilized in the genotyping PCR employing Q5^®^ High-Fidelity DNA Polymerase (M0491S New England BioLabs Gmbh, Frankfurt am Main, Germany) and the primer strategy summarized in Table 1. The fragment correspondent to canine SZT2 N-terminal and upstream region was then sequenced and used for the Tracking of Indels by Decomposition (TIDE) analysis (https://tide.nki.nl, accessed on 4 August 2021) to assess the spectrum and frequency of indels generated by our KO gene editing strategy. An indel size range of 20 bp and a *p*-value threshold < 0.001 were selected as parameters.

### 2.7. Total Cell Lysates and Immunoblotting

Cells were washed twice in ice cold 1x PBS, removed from the plate and centrifuged at 13,000× *g*, 4 °C. Cell pellets were resuspended in Lysis buffer (50 mM HEPES pH 8.0, 150 mM NaCl, 5 mM EDTA pH 8.0, 0.5% NP-40, 50 mM NaF, 10 µg/mL Leupeptin, 0.4 mM Pefablock, 1 µg/mL Pepstatin, 10 µg/mL Aprotinin, 0.5 mM PMSF, 1 mM Na_3_VO_4_) and lysed for 30 min on ice. Upon centrifugation at 13,000× *g* and 4 °C, cleared lysates were normalized and supplemented with urea buffer (2 M Urea, 50 mM Tris-HCl pH 8.0, 1% SDS, 1 mM EDTA pH 8.0, and 10 mM NaF) to a final concentration of 0.8 M Urea. SDS sample buffer was added, and the lysates were heated up to 60 °C for 10 min.

Lysates or other protein samples were separated by SDS-Polyacrylamide Gel Electrophoresis (PAGE).

Polyacrylamide gels were prepared consisting of a stacking (125 mM Tris pH 6.8, 4% Acrylamide/Bis solution (37:5:1), 6% glycerol, 0.1% SDS, 0.075% APS, and 0.1% TEMED) and a resolving gel (0.375 mM Tris pH 8.8, 7–15% Acrylamide/Bis solution (37:5:1), 0.1% SDS, 0.05% APS, and 0.05% TEMED). For SZT2 detection, we ran 5% polyacrylamide resolving gels that were allowed to run until the 140 kDa reference band reached the front. All SDS PAGE gels were run in 192 mM glycine, 25 mM Trisma Base, 0.1% SDS. After separation, the proteins were wet transferred onto ‘Amersham™ Protran™0.2 µm NC’ nitrocellulose membranes (GE10600002 Sigma-Aldrich Handels Gmbh, Vienna, Austria) at constant 70 V for 3 h for proteins with molecular weight higher than 300 kDa, or 2.5 h for proteins of a smaller size. The wet transfer buffer contained 25 mM Tris, 192 mM glycine, 0.1% SDS, and 20% methanol (*vol/vol*), adjusted to pH 8.3. Membranes were subsequently blocked in 3% BSA (fraction V), 1 mM EDTA, 0.05% Tween20, and 0.02% NaN_3_, and probed with the antibodies mentioned in Table 2. The antibody washings were performed in 100 mM NaCl, 50 mM tris-HCl pH 8.0, and 0.1% Tween-20. Finally, the ECL signal was detected and recorded with ‘Fusion FX EDGE’. Images were processed in ‘Inkscape 1.1’.

### 2.8. Immunofluorescence and Microscopy

Cells treated as indicated in Methods 2.1 were grown on glass coverslips. Upon starvation, cells were washed with ice cold PBS and fixed in methanol. Cells were then blocked and permeabilized in blocking buffer (150 mM NaCl, 10 mM pipes pH 6.8, 5 mM EGTA, 5 mM glucose, 5 mM MgCl_2_, 2% gelatin, 50 mM NH_4_Cl, 0.05% saponin (S-1252 Sigma-Aldrich Handels Gmbh, Vienna, Austria), and 5% BSA fraction V (8076.5 Roth, Lactan Vertriebs Gmb, Graz, Autria) for 45 min at RT. Slides were subsequently incubated with primary antibodies in blocking buffer for 1.5 h, at RT and washed 6 times with quenching and washing buffer (150 mM NaCl, 10 mM pipes pH 6.8, 5 mM EGTA, 5 mM glucose, 5 mM MgCl_2_, and 50 mM NH_4_C). Cells were incubated with secondary antibodies in blocking buffer for 1 h at RT in the dark and washed 6 times with quenching and washing buffer. Coverslips were then mounted in ‘ProLong™ Diamond antifade mountant’ (P36965 ThermoFisher, Life Tecnologies Austria Zweigniederlassung, Vienna, Austria). Epifluorescence images were acquired using a 63× oil immersion objective (NA 1.4) and the ‘Axio Imager M1 microscope’ (Carl Zeiss, Jena, Germany) equipped with a SPOT Xplorer, Visitron Systems camera. Images were reformatted to TIFF using ImageJ and adjusted for brightness and contrast using Photoshop 2020, Adobe, Mountain View, CA, USA.

Scanning EM was used for studying cell surface patterns according to standard protocols [49].

### 2.9. Cell Diameter Analysis

Cell size was measured after 3 h of starvation followed by 3 h of restimulation. Cells were starved and stimulated for amino acids and growth factors or starved for amino acids only as described above (2.1). When applicable, cells were treated with 330 nM Torin (Cay10997-10 Cayman Chemical, Ann Arbor, MI, USA). Upon treatment, cells were trypsinized and resuspended in medium of the corresponding conditions. The cell diameter was determined using an automated cell counter (Countess Automated Cell Counter, Invitrogen, Life Tecnologies Austria Zweigniederlassung, Vienna, Austria) following the manufacturer’s protocol. Three independent biological replicates were performed per cell line and condition. In each replicate the cell suspension was measured at least four times to assure that a minimum of 45,000 viable cells were counted. We only counted cells with a diameter in the range of 8 to 30 µm. Each measurement comprised three counting cycles. In each cycle, the instrument counted the number of cells with a given diameter (intervals of 0.05 µm ranging from 0 to 50 µm). In a subsequent analysis, we calculated and plotted the frequency data for each replicate with a bin size of 1 µm (cell diameter). In addition, the cell diameter with the highest frequency (peak cell diameter) per replicate was computed.

### 2.10. Statistical Analysis

The numerical results of the Western blot analysis are reported as mean + SEM. All remaining data shown as bar charts are presented as mean ± SD. For comparing more than two groups, we used either a two-way ANOVA with a subsequent Holm–Šídák correction for multiple testing or a nonparametric Kruskal–Wallis test with a subsequent multiple comparison Dunn’s test as indicated in the figure legend. The data were analyzed with GraphPad Prism (v 9.2.0, GraphPad Software, San Diego, CA, USA). Differences were considered statistically significant for *p*-values below 0.05; *p*-values are depicted in the charts.

## 3. Results

### 3.1. Establishment of the SZT2 Interactome

**Figure 1:**
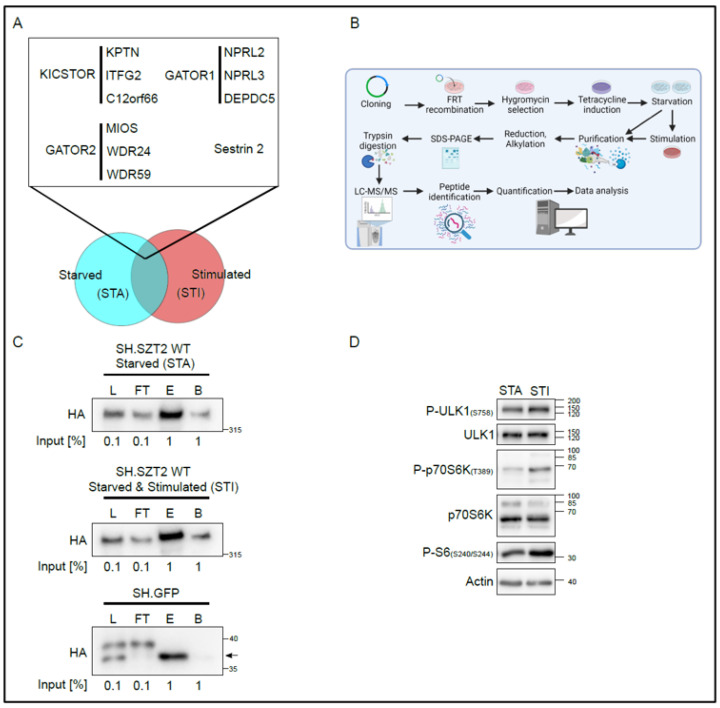
Establishment of the SZT2 interactome. (**A**) Graphical representation of workflow and choice of conditions. Published SZT2 binding partners associate with the protein independently of the physiological conditions, and we assume other interactors might also do so. In addition, SZT2 might bind to certain components under specific physiological conditions, e.g., starved (STA) and stimulated (STI). (**B**) Methodological workflow to decipher the SZT2 interactome under catabolic and anabolic conditions via affinity purification coupled to mass spectrometry. Image created with BioRender.com. (**C**) Quality control of the affinity purification fractions. An aliquot of specific fractions collected along the purification protocol was analyzed by Western blotting against HA; L, Lysate; FT, Flow through; E, Eluate; and B, Beads. (**D**) Lysates of a representative starved (STA) or starved and stimulated (STI) biological replicate were run on SDS-PAGE and analyzed by immunoblotting.

As SZT2 is a largely understudied mTORC1 regulator, we measured its interactome under different metabolic conditions (Figure 1A).

Our objectives were two-fold: first, we wanted to extensively chart the SZT2 interactome, assuming that SZT2 might be binding other proteins beyond the previously identified KICSTOR, Sestrin 2, GATOR1, and GATOR2 components. Second, although the known interactors associate with SZT2 independently of amino acid availability, SZT2 was found to be specifically required for mTORC1 inactivation upon amino acid depletion. Hence, we assumed that SZT2 might associate with yet unknown partners under different physiological conditions. Therefore, we decided to test conditions that would be linked to its canonical function but would be broad enough to elucidate novel roles of SZT2. As such, we determined the interactome under catabolic/starved conditions (STA), in which we depleted cells from growth factors and amino acids for 1 h or anabolic/stimulated conditions (STI), in which we restimulated the cells with both amino acids and growth factors for 20 min.

For the proteome analysis, schematically represented in Figure 1B, we generated a HEK293 Flp-In™ T-Rex™ cell line, inducibly expressing SZT2 tagged at the N terminus with STREP and HA. The choice of cell line was based on several factors, which we list here. First and foremost, although SZT2 mutations are causative for DEE, a disease with profound brain associated phenotypes, cortical neurons are not suitable for comprehensive biochemical studies. As such, the cell line of choice was a compromise. HEK293 were shown to express the neurofilament subunits NF-L, NF-M, NF-H, and α-internexin [50], endogenous β2-adrenergic receptors and βadrenergic receptor kinase 1 [51,52], sphingosine-1-phosphate receptors, somatostatin receptor subtype SSTR2, P2Y1, and P2Y2 receptors [53,54], and thyrotropin releasing hormone receptor [55], all of which are expressed in neuronal cells. Although derived from kidney tissue, HEK293 cells exhibited characteristics of immature neurons. In addition, most of the cell biology work reported on SZT2 to date has been performed using HEK293 [3,4] and we therefore decided to use this cell line to compare our results with previous data. Finally, we have successfully used HEK293 Flp-In™ T-Rex™ based tandem affinity purification in the past as a tool to decipher the proteome of other mTORC1 regulators [34,48].

For the subsequent proteome analysis, we followed an affinity purification coupled to mass spectrometry approach, which is schematically represented in Figure 1B. In brief, after total cell lysis, the induced baits (either SH.SZT2WT or SH.GFP) were subjected to STREP purification.

The large size of SZT2 and its association within the SZT2-orchestrated GATOR (SOG) complex (KICSTOR-GATOR1-GATOR2), whose molecular weight has been reported to peak at around 1.06 MDa [4], represented a technical challenge. We therefore altered our previous purification protocol [34,48] so as to compensate for the considerable size of the SZT2 containing complexes. With these improvements, we could retrieve SH.SZT2 in the eluates of both starved, or starved and stimulated cells (Figure 1C). As a control, we used the previously generated HEK293 Flp-In™ T-Rex™ expressing SH.GFP, and purified the bait under the same conditions.

To control the treatments performed in each of the six biological replicates per condition, we tested an aliquot of the respective lysates for the phosphorylation of the p70S6K at threonine 389, a bona fide substrate of mTORC1 [56,57] (Figure S1B). In all starved replicates, a reduction in the phosphorylation of p70S6K-T389 could be observed that increased upon stimulation. A more thorough analysis of the treatments is presented for one representative replicate in Figure 1D. Under starved conditions, there was a reduction in the phosphorylation of ULK1 at serine 757 [58,59], of the phosphorylation of p70S6K at threonine 389 and of S6 at serines 240/244, compared with stimulated conditions. In addition, we have successfully purified the baits in each of the biological replicates (Figure S1C), confirming the reproducibility of our purification protocol.

### 3.2. SZT2 Interactome Analysis

**Figure 2:**
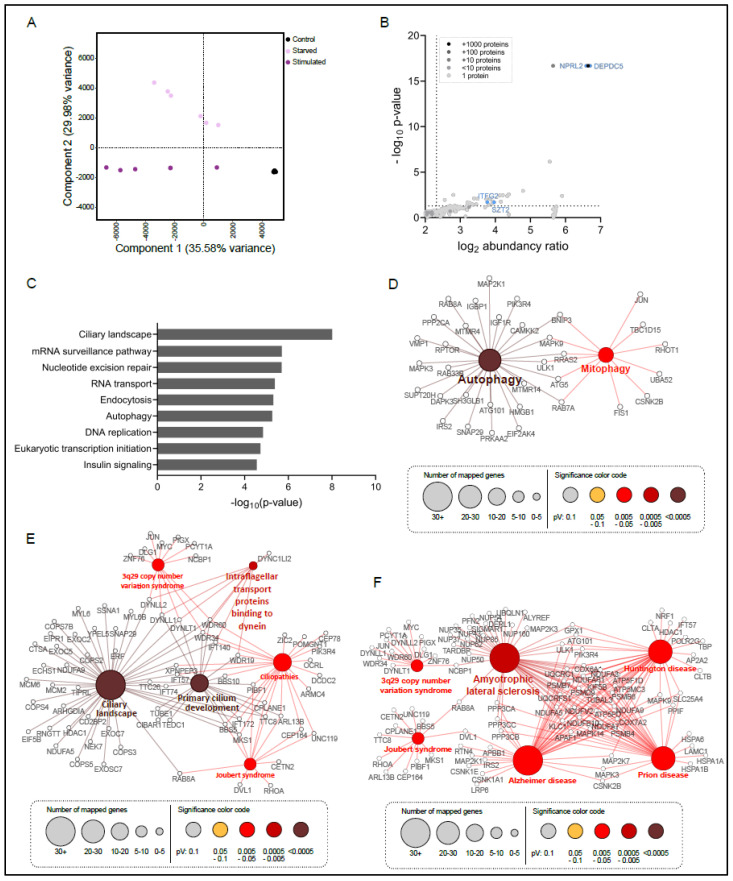
Characterization of the SZT2 interactome. (**A**) Principal component analysis (PCA) of the abundancies of all proteins identified in the interactome of SZT2 under starvation (light violet) or stimulation (violet) and of the GFP control (black). (**B**) Zoom in of volcano plot of all identified proteins interacting with SZT2 or the GFP control (see Figure S2A for whole volcano plot). The gray color scale depicts the number of proteins that have the same abundance and *p*-value. Proteins significantly binding to SZT2 have a *p*-value below 0.05 and an abundance ratio of 5 over the GFP control (within dotted lines). Representative components of KICSTOR and GATOR1 complexes are depicted in blue labeled with their protein names. (**C**) Summary of Top9 processes (KEGG pathway and WikiPathway) associated with proteins binding with high affinity to SZT2. Relevant processes were selected based on their −log10 (*p*-value). Maps of (**D**) autophagy, (**E**) cilia-associated terms, (**F**) neurological disorder-associated terms, with the depiction of proteins, which have a significantly higher affinity to bind to SZT2 than to the GFP control. The node size correlates with the number of mapped proteins per term and the node color with the significance of this term.

Processing of the SZT2 interactome data set resulted in the identification of 4818 proteins in total (ProteomeXchange dataset identifier PXD027662). To evaluate the data quality, we performed a principal component analysis (Figure 2A).

The six biological replicates of the GFP control clustered together and were clearly separated from the two independent STZ2 interactome clusters under starved or starved and stimulated conditions. The first component and the second component accounted for 35.58% and 29.98% of the summative variance, respectively. To identify specific SZT2 interactors, we carried out a statistical analysis of the proteins detected in the SH.SZT2 interactome performed under starved or under stimulated conditions, versus the SH.GFP control (two-sample analysis, Student’s *t*-test, *p*-value 0.05). We identified 1448 proteins with a *p*-value ≤ 0.05 and a fold-change over the GFP control ≥ 5 (Figure 2B and Figure S2A). Rewardingly, among the significantly regulated proteins we found the SZT2 bait and bona fide interactors such as ITFG2 (KICSTOR), NPRL2, and DEPDC5 (GATOR1) [3,4]. In addition, we detected several proteins with a clearly reported role in lysosomal mTORC1 signaling including RAPTOR, LAMTOR2, and LAMTOR3, and the vATPase.

The analysis of the nine most enriched processes associated with SZT2 interactors (Figure 2C), revealed terms such as endocytosis, autophagy, and insulin signaling. Unexpectedly, the most significant term found was the ciliary landscape. The importance of this finding will be discussed later. The comprehensive representation of all significantly enriched processes can be found in Figure S2B. Considering the thematic clusters of the individual processes, metabolic signaling and metabolism, regulation of transcription and translation, DNA repair, and cancer associated proteins, clearly appear as key regulated processes. Within the metabolic signaling cluster, SZT2 interacted with both mTOR and AMPK components.

In particular, we were surprised that, of the 1448 interacting partners, we found 241 involved in four specific processes, namely the development of neurological diseases (93), cilia and ciliogenesis (72), endocytosis (42), and autophagy (34) and decided to look at these in more detail (Figure 2D–F).

The first process that we focused on was autophagy (Figure 2D). Within this group we found bona fide mTORC1 related proteins such as RAPTOR and the mTORC1 substrate ULK1 (a key autophagy regulator), but also the Rab GTPases 8A, 7a, and 33B. The interactome also contained ATG101, an essential component of the autophagy-initiating ULK1 complex, ATG5, and the SNARE SNAP29 that is required for autophagosome/lysosome fusion [60,61]. We also found proteins specifically involved in mitophagy, such as BNIP3, required for mitochondrial priming prior to autophagic recognition [62], and Fis1, a key regulator of PINK1/Parkin-independent mitophagy [63]. Within the ciliogenesis cluster (Figure 2E), the most significant terms were cilium development and ciliary landscape. The presence of both terms caught our attention as it might indicate the involvement of SZT2 at different stages of ciliogenesis. We also found a significant number of hits (6%) playing a role in ciliopathies, in particular in 3q29 copy number syndrome and Joubert syndrome. Both diseases link cilia to neurogenesis and are therefore also present within the neurological disorders’ clusters (Figure 2F). This group also includes Huntington’s disease (HD), Prion disease, Alzheimer’s disease (AD), and Amyotrophic Lateral Sclerosis (ALS). Of note, most of these diseases are linked to neuronal degeneration, and some include cognitive impairment and epilepsy, features that are also observed in DEE.

### 3.3. Metabolic State Alters the SZT2 Interactome

**Figure 3:**
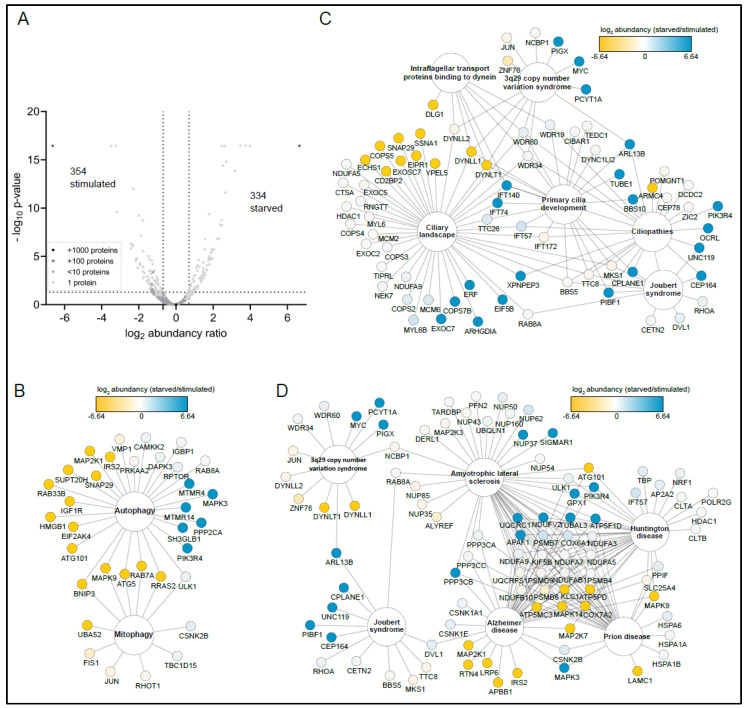
Interactome analysis revealed SZT2 interactors that preferentially bind upon starvation or stimulation. (**A**) Volcano plot of all identified proteins interacting with SZT2 with the abundancy ratio of interacting proteins upon starvation versus stimulation. The gray color scale depicts the number of proteins that have the same abundance and *p*-value. Proteins binding with a higher affinity to SZT2 upon starvation have a *p*-value below 0.05 and an abundance ratio of 1.5 over proteins binding upon stimulation (within right dotted lines). Map of (**B**) autophagy, (**C**) cilia-associated and (**D**) neurological disorder-associated terms, depicting identified proteins found in the SZT2 interactome. The color code shows the log2-abundance ratio of the single proteins with proteins binding SZT2 upon starvation colored in blue and upon stimulation colored in yellow.

To address whether SZT2 might associate with specific partners depending on the physiological status, we analyzed the differential proteome under starvation and restimulation conditions. Our analysis identified 686 proteins fitting our selection criteria (*p*-value ≤ 0.05 and a fold-change ≥ 1.5). Among these, we found 334 enriched in the starved and 352 in the restimulated cells (Figure 3A).

We then examined whether proteins present in the three selected processes (autophagy, ciliogenesis, and neurological disorders) were detected under specific conditions (Figure 3B–D). Surprisingly, 45% of the autophagy proteins retrieved in the interactome were found only under stimulated conditions (Figure 3B) with a small subset (17%) corresponding to MTMR4, MAPK3, MTMR14, SH3GLB1, PP2CA, and PIK3R4, exclusively present in starved cells. Concerning the mitophagy cluster, all the proteins were either present in both or found exclusively under stimulated conditions. Out of the proteins associated with ciliopathies and cilia development, 81% were either not differentially present or specifically found under starved conditions (Figure 3B). In contrast, 18% of all proteins associated with the ciliary landscape were found only in stimulated cells. For neurological disorders (Figure 3D), we detected 51% of hits in both conditions (e.g., HD or ALS clusters). A total of 23 interactors were common to the AD, prion, HD, and ALS clusters. These hits did not show any preference for treatment conditions: some were found under starved, some under stimulated, and some under both. The AD cluster included five specific hits detected under stimulated conditions: MAP2K1, RTN4, LRP6, APBB1, and IRS2. In contrast, the Joubert syndrome cluster contained five hits found under starved conditions, CPLANE1, UNC119, PIBF1, and CEP164. Taken together, the significant number of hits associated with ciliogenesis, autophagy, and neuronal functions, and the fact that many of them associate with SZT2 under stimulated conditions, led us to hypothesize that SZT2 might participate in biological processes beyond the detection of amino acid depletion and subsequent mTORC1 inactivation.

We then validated the interactome results by probing SH.SZT2 eluates for the presence of a selected repertoire of proteins representing the different clusters (Figure S3A). As quality controls, SZT2 and KPTN were found on both SH.SZT2 eluates, but not in the control eluate from SH.GFP. In addition, we detected the presence of RAPTOR and TSC2 proteins in SZT2 eluates. To probe for an interaction with ciliary components, we analyzed the eluates for the presence of the centriolar coiled-coil protein of 110 kDa (Cp110), which can suppress the formation of primary cilia [64], and γ-tubulin, which is found at the base of primary cilia at the microtubule organizing center (MTOC) [65]. We found both exclusively present in SZT2 eluates. As bioinformatic analysis showed a prominent link between SZT2 and neurogenesis, we manually checked the 1448 proteins that comprise the SZT2 interactome for possible neuronal related proteins that might have escaped our initial analysis. We found FMR1/FMRP as an interactor of SZT2 and we validated this interaction by Western blotting (Figure S3A,B). Mutations in FMRP are causative for the neurological disease fragile X syndrome. This condition includes behavioral alterations, intellectual disability, poor language development, and seizures, sharing many of the phenotypes observed in patients with pathogenic mutations in SZT2 [66].

### 3.4. Characterization of SZT2 KO Cells

As tools to elucidate SZT2 dependent functions, we generated knockouts (hereafter KO) of the gene in HEK293 and HEK293 Flp-In™ T-Rex™ cells via CRISPR/Cas9. The screening strategy and the genotyping results can be seen in Figure S4A. The first allele of HEK293 KO cl.128 had a 396 bp deletion of the 5’UTR and the entire exon 1 that prevented protein production. The second allele had an 8 bp deletion within exon 1 leading to frameshift and nonsense-mediated decay (NMD). HEK293 Flp-In™ T-Rex™ KO cl.63 had two SZT2 alleles that sustained in the first exon a 5 bp deletion or an 8 bp deletion, respectively, both leading to frameshift and NMD. Clone 84 had one allele with a 303 bp deletion plus 3 bp insertion that removed the 5’UTR and exon 1. The second allele underwent a 1 bp duplication that triggered NMD. No untargeted/wild type sequence was detected in either of the clones.

**Figure 4:**
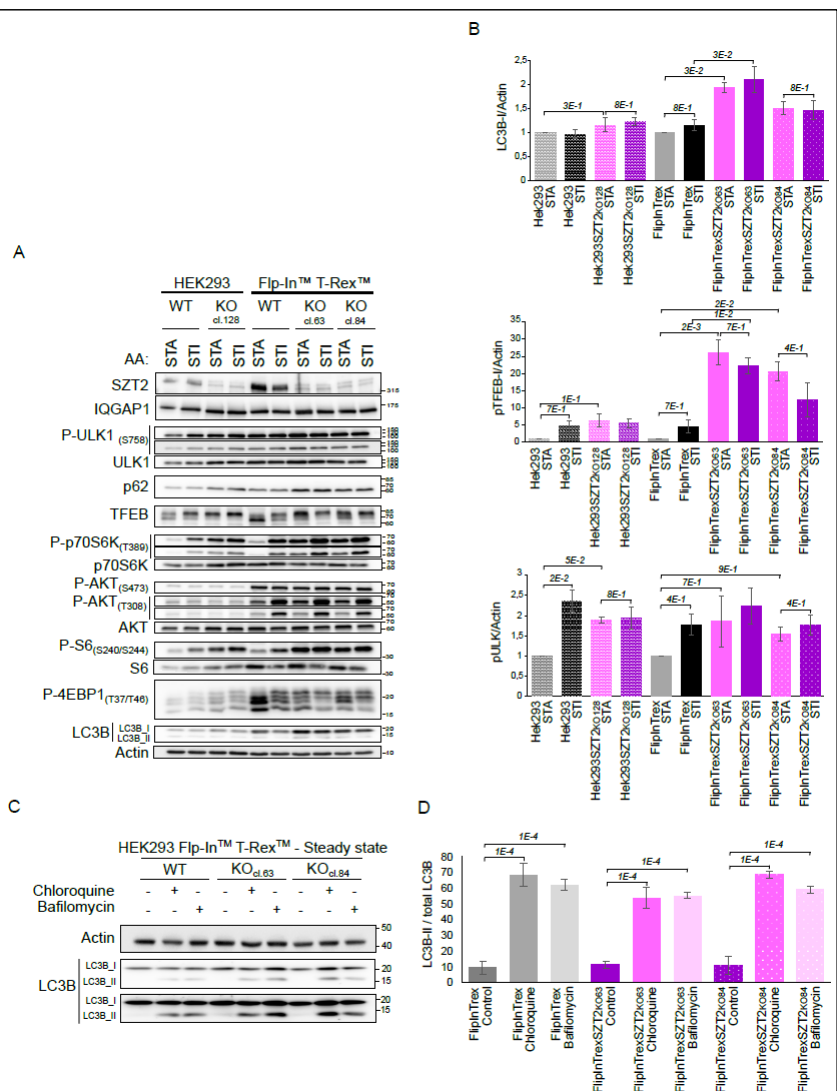
Characterization of the SZT2 KO cells under amino acids starvation and stimulation conditions. (**A**) Wild type and SZT2 KO cells were starved for amino acids for 1 h followed or not by restimulation for 20 min. The corresponding lysates were run on SDS-PAGE and analyzed by immunoblotting. SZT2 was detected on a separate membrane with IQGAP1 detection as a loading control. Data are representative of three independent experiments. (**B**) Relative quantification of the expression of selected proteins detected in A. Graphics display mean + SEM. (**C**) Wild type and SZT2 KO cells were treated with either Chloroquine or Bafilomycin for 6 h. Obtained lysates were run on SDS-PAGE and analyzed by immunoblotting. Data are representative of three independent experiments. (**D**) Relative quantification of expression of LC3B-II versus total LC3B. Graphics display mean + SEM.

We initiated our functional characterization by testing the generated KO cell lines and their wild type controls under amino acids starvation (1 h) and restimulation (20 min) conditions (Figure 4A).

The signal detected with the SZT2 antibody was stronger in the parental wild type cells than in the SZT2 KO cells, where we detected two weak bands. We hypothesize that these are antibody cross-reactions, as our genotyping results indicate that all three cell lines are KOs. The differences in endogenous SZT2 expression levels between HEK293 and HEK Flp-In™ T-Rex™ WT are due to the nature of the respective cell lines. Importantly, the control cell lines reacted to starvation and restimulation treatments by regulating the phosphorylation of p70S6K, S6, and 4EBP1 [67,68]. Although we did not probe with a phosphosite specific antibody, we saw a shift in the total TFEB blot upon stimulation, indicating phosphorylation of the protein [69,70]. In contrast, SZT2 KOs showed a high level of phosphorylation of the mentioned mTORC1 substrates under starved conditions. Encouragingly, these results replicate previously published data and confirm the involvement of SZT2 in amino acid sensing [3,4]. Of note, although mTORC1 activity in starved SZT2 KOs was comparable to that of stimulated control cells, we observed that the KOs could further boost mTORC1 upon stimulation. This indicates that SZT2 KOs maintain, to a certain extent, the capacity to sense the presence of amino acids (visible at the lower exposure blot for P-p70s6K, TFEB, and P-4EBP1). The regulation of ULK1 phosphorylation at S757, was not as pronounced as for the remaining mTORC1 substrates, with small changes between different conditions and cell lines. To explore whether mTORC2 or the IRS/PI3K pathway contribute to the observed mTORC1 hyperactivity phenotype under SZT2 deficiency, we immunoblotted for phosphorylation of AKT at S473 and T308. No difference between the WT and KO cell lines was observed for S473, the mTORC2 site [71]. In contrast, stimulation with amino acids led to an increase in the PDK1 dependent phosphorylation of AKT at T308. This regulation, however, was only visible in HEK293 Flp-In™ T-Rex™ and independent of the genotype.

Taking into consideration the vast number of autophagy hits present in the interactome, we decided to additionally probe the membranes for LC3B and p62. Surprisingly, we observed sustained or higher levels of LC3B-I, the cytoplasmic version of the protein, and of p62 in the SZT2 KOs, regardless of the experimental conditions. This effect was most pronounced with HEK293 Flp-In™ T-Rex™ KO cl.63. It is well established that TFEB and ULK1 are mTORC1 substrates that control autophagy [58,72]. Counterintuitively, we observed increased phosphorylation of TFEB preventing its nuclear translocation, sustained phosphorylation of ULK1, and increased LC3B-I levels in SZT2 KO. This could be a consequence of upregulated transcription and/or translation, of defective LC3B lipidation or of impaired phagolysosomal degradation. As the effects on LC3B and p62 were independent of the conditions tested, we decided to address autophagic flux under steady state conditions (Figure 4C). For this purpose, we treated the cells with either Chloroquine or Bafilomycin for 6 h. Both inhibitors led to an accumulation of the LC3B-II form in both wild type and SZT2 KO cells, indicating normal LC3B conversion. The elevated LC3B-I levels associated with normal LC3B-I to -II conversion in SZT2 deficient cells under sufficient nutrient provision indicate induction of basal autophagy in an mTORC1 hyperactive context.

Several observations lead us to speculate that SZT2 might be linked to selective autophagy. First, we detected the presence of a substantial number of autophagy regulators in the SZT2 interactome under stimulated conditions (Figure 3C). Second, we detected mitophagy regulators in the interactome. Third, we observed sustained or increased levels of LC3B-I in both starved and stimulated conditions (Figure 4A). To address this hypothesis, we queried the SZT2 interactome for the presence of selective autophagy receptors/regulators (see Figure S3B). Notably, the SZT2 interactome included NUFIP1, USP14, BNIP1, TEX264, and Pex14 (>5 fold-change over the control, *p* < 0.05), respectively, representing ribophagy, mitophagy, ER-phagy (reticulophagy), and pexophagy receptors/regulators [73,74,75,76,77,78,79]. Moreover, galectin 3 (LGALS3) a protein that, together with TRIM16, coordinates the recognition of damaged endomembranes by the autophagy system [80], was also present in the SZT2 interactome.

### 3.5. Effects of mTORC1 Inhibitors on SZT2 KO Cells

**Figure 5:**
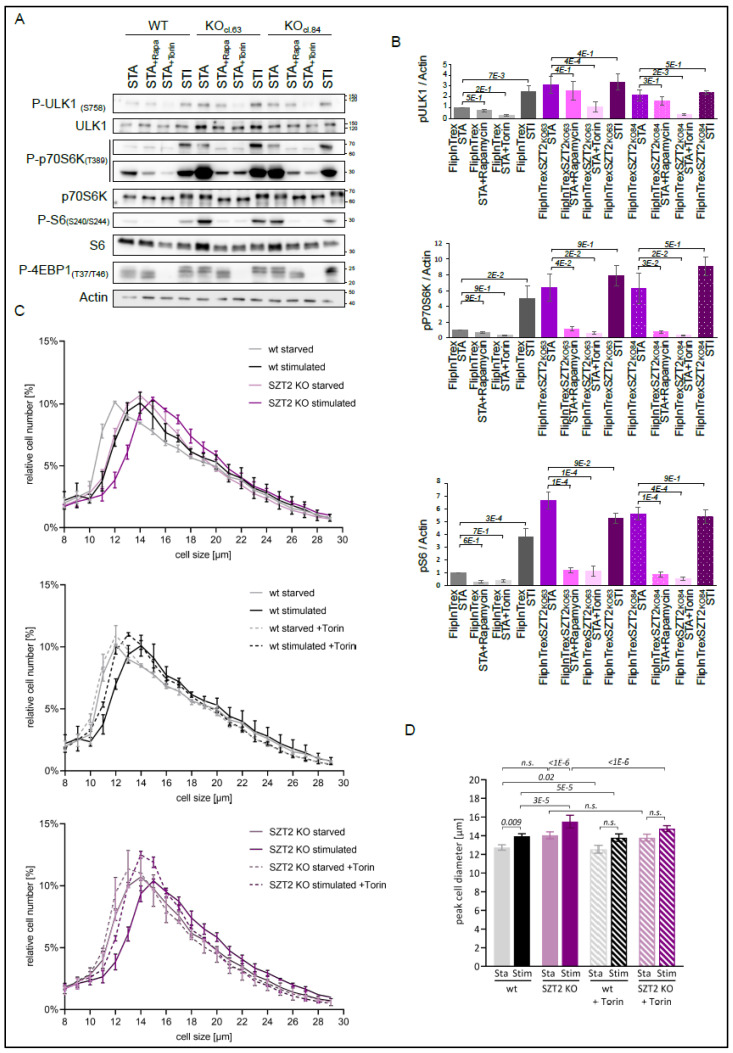
Torin and Rapamycin reduce the mTORC1 hyperactivity seen in SZT2 KO cells. (**A**) Wild type and SZT2 KO cells were starved for amino acids for 1 h followed or not by restimulation for 20 min. In addition, cells were treated with either Rapamycin or Torin during starvation. Obtained lysates were separated by SDS-PAGE and the presence of the indicated proteins determined by immunoblotting. Data are representative of three independent experiments. (**B**) Analysis of the regulation of selected proteins detected in (**A**). Graphics display the mean + SEM. (**C**) Control and SZT2 KO cells were starved for amino acids for 3 h and restimulated for another 3 h prior to harvesting. The cell diameter of three biological replicates per cell line and condition were analyzed. Graphics represent the cell size of at least 45,000 viable cells per condition plotted against their relative number ± standard dev. (**D**) Quantification of the peak cell diameter of each cell line analyzed in (**C**). Graphic represents the peak diameter in the different conditions ± standard dev. The statistical significance was analyzed using a nonparametric Kruskal–Wallis test and subsequent Dunn’s test with the *p*-value depicted in italics.

To further investigate whether the mTORC1 signaling defects found in SZT2 KO cells could be restored by treatment with mTORC1 inhibitors, we treated the cells under amino acid starvation with either Rapamycin or Torin [81,82] (Figure 5A,B).

Control cells showed reduced phosphorylation of all mTORC1 substrates tested under amino acid starvation that could be increased by restimulation. Treatment with Rapamycin or Torin further decreased the already low levels of phosphorylation of the substrates found under starved conditions. Corroborating the results from Figure 4A, SZT2 KOs showed increased phosphorylation of p70S6K, S6, and 4EBP1 under starved conditions when compared with the wild type control. Treatment with Rapamycin and Torin reduced this phosphorylation to the levels of the starved control. In accordance with previous publications, Torin had a stronger effect on the phosphorylation of ULK1 and 4EBP1 than Rapamycin [83,84]. This effect was independent of the genotype.

The cellular processes that regulate cell size are, in part, controlled by mTOR activity [85,86]. As such, cell size determination has become an easy tool to assess cellular metabolism, and determine the relative contribution of different mTORC1 regulators. It has been previously reported that SZT2 deficiency in HEK293T cells leads to increased cell size under steady state conditions [4]. Building on this knowledge, we determined the diameter of SZT2 KO cells under different metabolic conditions. We first performed the experiment solely under amino acid starvation and restimulation (Figure 5C,D). Control cells reacted by significantly increasing their diameter under stimulated conditions. Despite being larger, SZT2 KOs still reacted to the presence of amino acids by increasing their diameter even further. Treatment with Torin shifted the frequency distribution profile of the wild type stimulated cells to lower values, so that no significant difference could be found between starved and stimulated cells. The same effect could be observed for the SZT2 cells upon Torin treatment.

In parallel, we also determined the diameter of SZT2 KO and control cells under starvation of amino acids and growth factors, and then restimulation (Figure S5A,B). Both control and SZT2 KOs reacted to the conditions, although SZT2 KOs were, in general, larger in diameter. Interestingly, under these stimuli, the treatment with Torin did not alter the diameter profiles of the control cells. As a consequence, the stimulated cells remained larger than the starved ones. Torin treatment of the SZT2 KO cells shifted the frequency distribution profiles of the curves slightly to the right. Under starved conditions this shift was less pronounced, and the mean peak diameter was not significantly changed upon addition of Torin. In contrast, under restimulated conditions, we could detect a statistically significant decrease in the peak cell diameter upon Torin treatment.

Taken together, our observations suggest that mTORC1 inhibitors, in particular Torin, can be used to circumvent mTORC1 hyperactivation and the shift into anabolism found in SZT2 KO cells. Torin also reverted the increased cell size of SZT2 ablated cells.

### 3.6. SZT2 in Ciliogenesis

**Figure 6:**
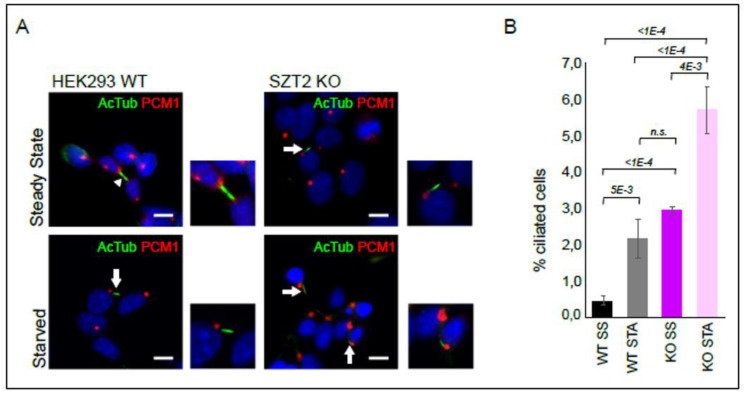
Ciliogenesis in SZT2 KO cells. (**A**) Control Hek293 Flp-In™ T-Rex™ cells and SZT2 KO cl.63 were either left untreated or starved for growth factors for 72 h to induce ciliogenesis. Cells were then fixed and stained with antibodies against acetylated tubulin (green) and PCM1 (red). The nucleus of the cells was counterstained with Hoechst. Arrow head points to a midbody that is also enriched in acetylated tubulin. Arrows indicate cilia. Scale bar 10 µm. (**B**) Quantifications of (**A**) representing the percentage of ciliated cells found in each cell line and condition ± standard dev. Statistical significance was calculated using a nonparametric Kruskal–Wallis test with subsequent Dunn’s test and the italic numbers represent the *p*-value.

As described above, ciliary landscape was the most significantly regulated process in the SZT2 interactome (Figure 2C). Although HEK293 cells show reduced ciliary formation, they have been used before to study ciliogenesis [87]. We therefore compared the number of cilia in SZT2 KO vs. control cells (Figure 6).

**Figure 7:**
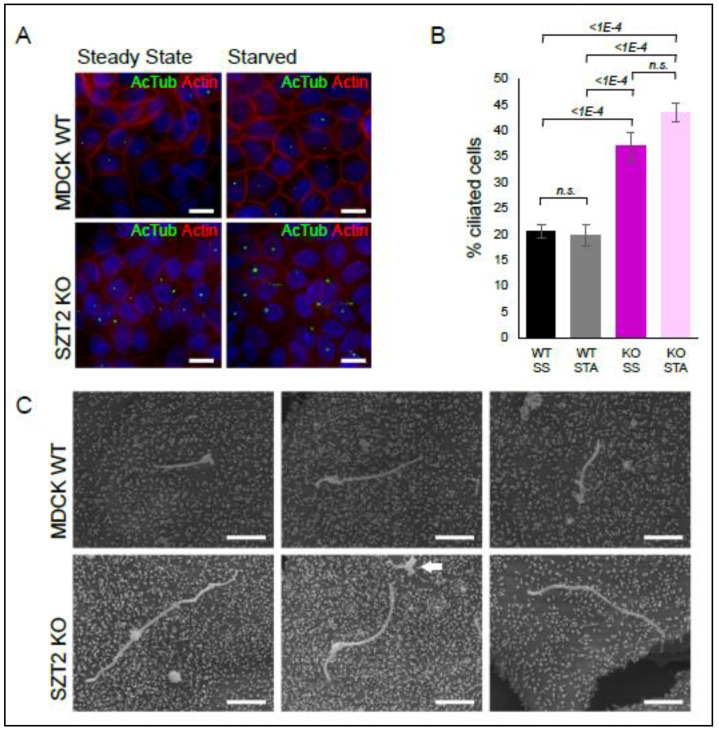
Ciliogenesis in MDCK SZT2 bulk KO cells. (**A**) Control MDCK cells and SZT2 bulk KO were grown to confluency and then either left untreated or starved for growth factors for 48 h. Cells were then fixed and stained with antibodies against acetylated tubulin (green) and Phalloidin (red). The nucleus of the cells was counterstained with Hoechst. Scale bar 10 µm. (**B**) Quantifications of (**A**) representing the percentage of ciliated cells found in each cell line and condition ± standard dev. More than 2000 cells per condition and replicate were counted. Statistical significance was calculated using a nonparametric Kruskal–Wallis test with subsequent Dunn’s test and the italic numbers represent the *p*-value. (**C**) Scanning EM of controls and MDCK SZT2 bulk KO cells. Three representative cilia from each cell line are shown. Arrow indicates shrunken cilia. Scale bar 2 µm.

Under steady state conditions, less than one percent of wild type cells showed cilia, assessed by the presence of a bundle of acetylated tubulin corresponding to the axoneme (green) emanating from the basal body, detected with a PCM1 antibody (red). Instead, under the same conditions, SZT2 KO had nearly 3% of ciliated cells. Prolonged growth factors starvation (72 h) lead to an increase in the number of ciliated cells but the trend remained: SZT2 KO cells showed a three-fold increase in ciliogenesis in comparison to controls. To corroborate these results, we decided to repeat these experiments in Madin–Darby canine kidney (MDCK) cells, a common model of ciliogenesis, and epithelial vesicular trafficking [88,89]. We generated a SZT2 bulk KO cell line with 80% editing of the wild type STZ2 alleles (Figure S6). All the detected indels corresponded to a 1 bp insertion within exon 1 predicted to result in frameshift and NMD. Control and SZT2 bulk KO MDCK cells were grown to confluency and then starved for amino acids and growth factors for 48 h or maintained in cultivation medium containing serum and amino acids for the same period of time (Figure 7).

The majority of the cilia observed were curled up and shrunken, most probably due to the fixation procedure [90]. For this reason, we counted all observed cilia, independent of their morphology. Supporting the data on HEK293, the percentage of ciliated cells was twice as high in the MDCK SZT2 bulk KO than in control cells (Figure 7A,B). We observed no gross differences between cells starved or maintained in full medium, that we attribute to the fact that all cells were brought to confluency beforehand. To appreciate the morphology of the cilia, we subjected the cells to scanning electron microscopy (Figure 7C). Cilia from the SZT2 bulk KO cells were in general longer than those of control cells.

## 4. Discussion

The interactome data presented here endorse previous findings classifying SZT2 as a regulator of amino acid sensing for mTORC1. We not only confirmed the interaction with KICSTOR and GATOR1 components, but also extended these findings by additionally detecting RAPTOR, TSC2, LAMTOR, and the vATPase, all of which are involved in lysosomal mTORC1 signaling. mTORC1 and AMPK are yin–yang regulators of cellular metabolism. While the former promotes anabolic processes, the latter inhibits them, and switches the metabolism to catabolic mode. The opposing synchronization of the two signaling pathways has been the subject of extensive research in the past that has uncovered bidirectional regulation [91]. Although AMPK has long been known to inhibit mTORC1, it was recently shown that mTORC1 inhibits AMPK to promote cell proliferation under nutrient stress. [92]. In light of the experimental setup chosen for the interactome analysis, with starvation (promoting catabolism) and stimulation (promoting anabolism) the presence of these components can be seen as an additional quality control. Moreover, we would like to emphasize that a number of processes regulated by SZT2 corresponded to interactions occurring under anabolic (stimulated) conditions, in contrast to the previously defined role as a suppressor of mTORC1 activity in the absence of amino acids (catabolic conditions).

The most important contribution of this comprehensive annotation of interactors was undoubtedly the clear association of SZT2 with ciliogenesis, autophagy, neuronal function, and neurodegeneration. In the following sections, we interpret our results based on published observations, and present an integrated model of potential SZT2 functions. Under starvation conditions, cells enter the resting state and activate autophagy to degrade cytoplasmic proteins and organelles to provide the nutrient blocks (i.e., amino acids) required to maintain basic cellular activity. This process is accompanied by two actions that increase the cell’s ability to recognize extracellular nutrients: (a) the general amino acid control (GAAC) pathway that upregulates amino acid transporters at the plasma membrane [93], and (b) the growth of primary cilia that provides a “scaffolding” location for many receptors, maximizing sensing and signal integration [94]. Therefore, the interaction of SZT2 with proteins involved in both ciliogenesis and autophagy may prove to be mechanistically relevant in the context of the disease we are studying.

Macroautophagy, frequently abbreviated as autophagy, is a generic process triggered by catabolic conditions. Cytoplasmic material and organelles become trapped within autophagosomes, which then fuse with lysosomes to deliver their contents for degradation. This system not only allows the recycling of cellular components, but also provides a source of energy to the cell [95]. Besides this originally described bulk catabolic process, there is scientific consensus about the existence of selective (macro) autophagy, where autophagosomes catch specific organelles or protein aggregates [96]. To clarify the terminology used, we distinguish between selective and non-selective autophagy. These processes can either take place at basal or under induced conditions (stress or starvation) [97].

Neurons are post-mitotic cells that cannot simply thin out toxic protein aggregates or damaged organelles by cell division [98]. As a consequence, basal autophagy assumes a prominent role in the development and functionality of the central nervous system. Indeed, basal autophagy is required for the maintenance of axonal homeostasis [99] and suppression of basal autophagy in neural cells causes neurodegenerative diseases in mice [100,101]. Autophagy was identified as a significantly regulated process in the SZT2 interactome, and most of the hits observed in this cluster were associated with SZT2 under anabolic conditions. Moreover, in SZT2 KO cells, we observed increased levels of LC3B-I and p62 present in both starved and stimulated conditions, accompanied by increased phosphorylation of TFEB and sustained phosphorylation of ULK1. We also addressed LC3 conversion in the KO cells and saw that it was not impaired. In agreement with our results, a 2018 case report analyzed dermal fibroblasts of a patient with compound heterozygous mutations in SZT2. Under transmission electron microscopy, these cells displayed abundant lysosomes filled with undigested membranes indicative of autophagy [102]. It remains unclear how basal autophagy is regulated under mTORC1 hyperactivation. We took a closer look at reports from TSC1 and TSC2 KOs, with bona fide mTORC1 hyperactivation, to see if a possible mechanism has been proposed in these cells. In contrast to MEFs, neurons with TSC2 reduction display increased levels of LC3B and p62. The observed autolysosome accumulation was triggered by AMPK dependent ULK1 activation [103]. A similar effect was seen in TSC1 deficient macrophages [58]. In contrast, a recent publication reported that renal tumors, lymphangioleiomyomatosis, and kidneys from TSC2 heterozygous mice display increased lysosomal biogenesis that occurs due to hypo-phosphorylation and nuclear translocation of TFEB [104]. In the SZT2 KOs we observed increased phosphorylation of TFEB, ruling out a contribution of this transcription factor. The possible participation of AMPK [105] will be addressed in upcoming work. Despite these open questions, our results clearly indicate a role of SZT2 in the regulation of basal autophagy.

Our interactome results also shed light on the type of autophagy that is regulated under basal conditions. In fact, one of the most surprising findings of our analysis has been the detection of mitophagy, ribophagy, ER-phagy (reticulophagy), and pexophagy receptors/regulators [73,74,75,76,77,78,79]. As an example, poly-ubiquitylated BNIP1, one of the proteins found in the SZT2 interactome, builds a bridge between mitochondria and autophagosomes by binding to the selective autophagy receptor p62 [79]. In line with our results, fibroblasts from patients with SZT2 mutations showed impaired mitochondrial respiratory activity associated with the accumulation of elongated and abnormally shaped mitochondria, suggestive of altered organelle dynamics [102]. Of note, altered mitophagy equilibrium has been associated with Alzheimer’s and Huntington’s diseases [98,106], that were also represented in the SZT2 interactome. As another example, the peroxisomal membrane protein PEX14 delivers the organelle to autophagosomes via direct LC3 interaction [107,108]. Although pexophagy can be triggered by many diverse stimuli [109], under basal conditions it determines the renewal rate of peroxisomes with an approximate half-life of 2 days [110]. Impaired peroxisomal homeostasis is associated with Zellweger syndrome, a disease that, similar to DEE, includes craniofacial dysmorphism and infantile epileptic seizures as characteristic features [111,112]. In addition, a strong interaction between mTORC1 and peroxisomal membrane proteins has been previously reported, including the localization of TSC1, TSC2, and Rheb at peroxisomes [5,111].

Fragile X syndrome (FXS) is a genetic nervous system disorder characterized in particular by cognitive impairment, autism, and seizures, caused by loss-of-function mutations in the fragile X mental retardation 1 protein (FMRP) [66]. FMRP is a ribosome associated RNA-binding protein that, in broad terms, acts as a translational repressor. Its regulatory effects on mRNA occurs at multiple levels: it controls splicing, mRNA stability, and transport [113,114,115]. RNA granule formation is regulated by RNA binding proteins such as FMRP and acts as a mechanism to locally suppress translation. Of note, granule formation has been linked to neurological diseases, and is often triggered as an adaptation to catabolic conditions [116]. Of utmost relevance for us is the work by Wyant et al., reporting that NUFIP1, also found in our SZT2 interactome, is a known binding partner of FMRP and functions as a ribosome receptor for starvation induced ribophagy [73]. Moreover, activation of autophagy corrected the spine structure, synaptic plasticity and cognition impairment observed in a mouse model of FXS [117]. In addition, FMRP modulates the gating activity of the sodium-activated potassium channel KCNT1 [118] and the presynaptic large-conductance calcium-activated potassium (BK) channels in hippocampal and cortical excitatory neurons, affecting their action potential [119]. We found FMRP in the SZT2 interactome, and confirmed this result by Western blot analysis. Moreover, the presence of RNA transport, mRNA surveillance, and ribosomal function, as regulated KEGG pathways in the SZT2 interactome, start to make sense if one considers an interaction between SZT2 and FMRP. In addition, we also found SZT2 associating with LAMTOR components. The LAMTOR complex contributes towards mTORC1, MAPK, and AMPK pathways and is therefore in an ideal position to integrate and promote the crosstalk between different signaling cascades. Interestingly, altered MAPK activation has also been described in FXS KO mice [120]. It is therefore plausible that the connections between SZT2, FMRP, and LAMTOR play a role in the development of both DEE and FXS. This is certainly a promising line of investigation that should be addressed in future work.

Last but not least, ciliogenesis was the most significantly regulated process identified in the SZT2 interactome. Within this manuscript, we also confirmed the association of SZT2 with ciliary proteins by Western blot. In 2013, two studies shed light on the interplay between starvation induced autophagy and primary cilia: first, Pamgliega et al. reported that nutrient removal triggers the relocation of autophagic components to the cilia base, a process that is required for maximal autophagy induction [121]. Second, Tang et al. demonstrated that starvation induced autophagic degradation of a ciliary protein, OFD1 (oral-facial-digital syndrome 1), at centriolar satellites, promotes primary cilium biogenesis [122]. Interestingly, basal autophagy seems to suppress ciliogenesis rather than promoting it [121]. Pampliega et al., proposed that basal autophagy does so by degrading proteins required for intraflagellar transport. Overall, the interplay between autophagy and ciliogenesis might involve different components and be dependent on the overall cellular context [123]. Given the role of primary cilia as sensory organelles in neurons, and the fact that altered expression of cilia genes is a risk factor for several neuropsychiatric disorders [124,125,126], some of which are associated with seizure phenotypes, the interaction of SZT2 with proteins involved in both cilia formation and autophagy may be of great importance in DEE. In addition, it is worth noting that the onset of functional glutamatergic synaptic activity coincides with the appearance of primary cilia, raising the possibility that the two processes are linked. [127].

There is accumulating evidence that primary cilia regulate mTORC1. In response to flow stress, kidney primary cilia downregulate mTORC1 through a FLCN-LKB1-AMPK mediated process [128]. Under this condition, cell size is also reduced [129]. In a reverse manner, cortical neurons of patients with somatic mutations in mTOR show reduced ciliation [130] and brains of TSC patients and central nervous system KO mouse models of TSC have reduced ciliation that can be restored by Rapamycin in vivo [131]. Interestingly, Rapamycin treatment does not affect ciliogenesis under basal conditions, whereas ablation of ATG proteins effectively promotes it [121].

Overall, there is ample evidence for intertwined regulation between ciliogenesis, autophagy, and neurogenesis/neuronal function. The fact that we observe all of the above as regulated processes in the SZT2 interactome opens up new conceptual views of how SZT2 mutations may possibly cause DEE. We propose that SZT2, through its strong interaction with the autophagic machinery, ensures optimal lysosomal degradation of autophagically sequestered cargoes under basal conditions. Moreover, the presence of SZT2 at the membrane of a mobile signal transduction platform (in this case, the lysosome) enables recruitment of the molecular machinery required for this purpose to cellular sites where selective degradation may be required. The apparent ability of SZT2 to regulate mTORC1 activity could be used as a mechanism to link the above processes to metabolic signaling. We hypothesize that this function may play a greater role in neurons than in other cells, including regulation of primary cilia and selective autophagy in axons/dendrites. Future work will be required to determine experimentally whether the proposed model holds true.

## Figures and Tables

**Table 1:** List of the primers used for the screening and genotyping PCR of the SZT2 KO cell lines. The primer names refer to the schematic representation of the screening PCR shown in Figure S4A.

Name	Sequence
Screening HEK293 Primer A	GCCTCGCCCCCCAGCCCAC
Screening HEK293 Primer B	CTCCGGCTCCGGGCGCTCCG
Screening HEK293 Primer C	TCGGGCCTTCCGGGCTGGGC
Genotyping HEK293 KO F	CATCTGTGAGCCTGGCTGTC
Genotyping HEK293 KO R	GAAGACTCGCCTGAGGTTGC
Genotyping MDCK KO F	CCCATCTCTTGCCAGGTGG
Genotyping MDCK KO R	AATGGCGACACCAATACTGGG

**Table 2:** List of the antibodies used.

Antibody	Species	Source	Identifier
β-Actin	Mouse	Cell Signaling Technology	3700S
Cp110	Rabbit	Abcam	ab243696
PCM-1 (Gln15)	Rabbit	Cell Signaling Technology	5259S
ULK1	Rabbit	Cell Signaling Technology	8054
phospho ULK1 (Ser757)	Rabbit	Cell Signaling Technology	6888
phospho p70S6K (Thr389)	Rabbit	Cell Signaling Technology	9234
p70S6K	Rabbit	Cell Signaling Technology	9202S
p62	Rabbit	Cell Signaling Technology	5114
LC3B	Rabbit	Cell Signaling Technology	2775
phospho S6 ribosomal Protein (Ser240/244)	Rabbit	Cell Signaling Technology	2215
S6 ribosomal Protein	Mouse	Cell Signaling Technology	2317
phospho 4E-BP1 (Thr37/46)	Rabbit	Cell Signaling Technology	2855 P
FMRP	Rabbit	Cell Signaling Technology	4317S
TFEB	Rabbit	Cell Signaling Technology	4240
TSC2	Rabbit	Cell Signaling Technology	4308
RICTOR	Rabbit	Cell Signaling Technology	2114Z
KPTN	Rabbit	Proteintech Group	16094-1-AP
LAMP-1	Mouse	Pharmingen	34201A
SZT2	Rabbit	Novus Biologicals	NBP1-89886
IQGAP1	Mouse	BD Biosciences	610612
α-Tubulin	Mouse	Developmental Studies Hybridoma Bank	12G10
RAPTOR	Rabbit	Cell Signaling Technology	2280
γ-Tubulin	Mouse	Sigma-Aldrich	T5326
phospho AKT (Ser473)	Rabbit	Cell Signaling Technology	4060S
phospho AKT (Thr309)	Rabbit	Cell Signaling Technology	13038S
phospho AKT (Thr308)	Rabbit	Cell Signaling Technology	9275
HA.11	Mouse	Biolegend	MMS-101R

## Data Availability

The mass spectrometry proteomics data were deposited to the ProteomeXchange Consortium (http://Proteomecentral.proteomexchange.org, accessed on 30 July 2021) [132] via the Proteomics identification database partner repository (PRIDE) [133] with the dataset identifier PXD027662.

## Supplementary Materials

The following are available online at https://www.mdpi.com/article/10.3390/cells10102711/s1, Figure S1: Quality control of the biological replicates used in the interactome analysis. (A) Lysates of each biological replicate were run on SDS-PAGE and analyzed by immunoblotting. Starved lysates (STA), starved and stimulated lysates (STI) (B). A fraction of each eluate was analyzed by Western blotting. The 27 kDa band corresponds to cleaved GFP. Membranes were probed with HA antibody. (C) Coomassie stained gels of the eluates were submitted to LC-MS/MS analysis. Individual replicates were split into 3 to 5 fractions prior to analysis; Figure S2: Overview of the SZT2 interactome. (A) Volcano plot of all identified proteins interacting with SZT2 or the GFP control. The gray color scale depicts the number of proteins that have the same abundancy and *p*-value. Proteins binding significantly to SZT2 have a *p*-value below 0.05 and an abundance ratio of 1.5 over the GFP control (within dotted lines). KICSTOR components are depicted in blue labeled with their protein names. (B) KEGG pathway and WikiPathway analysis of proteins binding SZT2 with a significantly higher abundance than the GFP control. The resulting terms are depicted as clusters with terms that are more general. The node size correlates with the number of mapped proteins per term and the node color with the significance of this term; Figure S3: Validation of selected interactors and manually selected hits from the interactome. (A) Eluates of affinity purified SH.SZT2WT and SH.GFP were run on SDS-PAGE and analyzed by immunoblotting. R = replicate. (B) Manually selected hits from MS analysis. STA = starved, STI = stimulated, ctrl = control, Ratio = abundance ratio in percentage, *p*-value = adjusted *p*-value, and n.a.= not available; Figure S4: Genotyping of the SZT2 KO cell lines. A schematic representation of screening PCR strategy and position of the respective primers is shown. The chromatograms of sequencing reactions from the two alleles of each SZT2 KO cell line are depicted. Differences to the reference sequence are stated. The localization of the alterations is annotated by a red line; Figure S5: Analysis of the cell diameter of SZT2 KO cells under starvation and restimulation with growth factors and amino acids. (A) Control and SZT2 KO cells were starved for amino acids and growth factors for 3 h and restimulated for another 3 h prior to harvesting. The cell diameter of three biological replicates per cell line and condition were analyzed. Graphics represent the cell size of at least 45,000 viable cells per condition plotted against their relative number ± standard dev. (B) Quantification of the peak cell diameter of each cell line analyzed in (A). Graphic represents the peak diameter in the different conditions ± standard dev. The statistical significance was analyzed using a nonparametric Kruskal–Wallis test and subsequent Dunn’s test with the *p*-value depicted in italics; Figure S6: Tracking of insertions and deletions (indels) in the MDCK SZT2 bulk KO cell line by decomposition (TIDE) analysis. (A) Plot of decomposition analysis with quantitative spectrum of indels around the Cas9 cut site.
