# Supplementary material for: The SZT2 Interactome Unravels New Functions of the KICSTOR Complex
Source: Cells. 2021 Oct 9;10(10):2711. doi: 10.3390/cells10102711 (PMC8534408; doi:10.3390/cells10102711)
Supplement: Supplementary file 1 [file cells-10-02711-s001.zip › cells-1361027-supplementary.pdf]

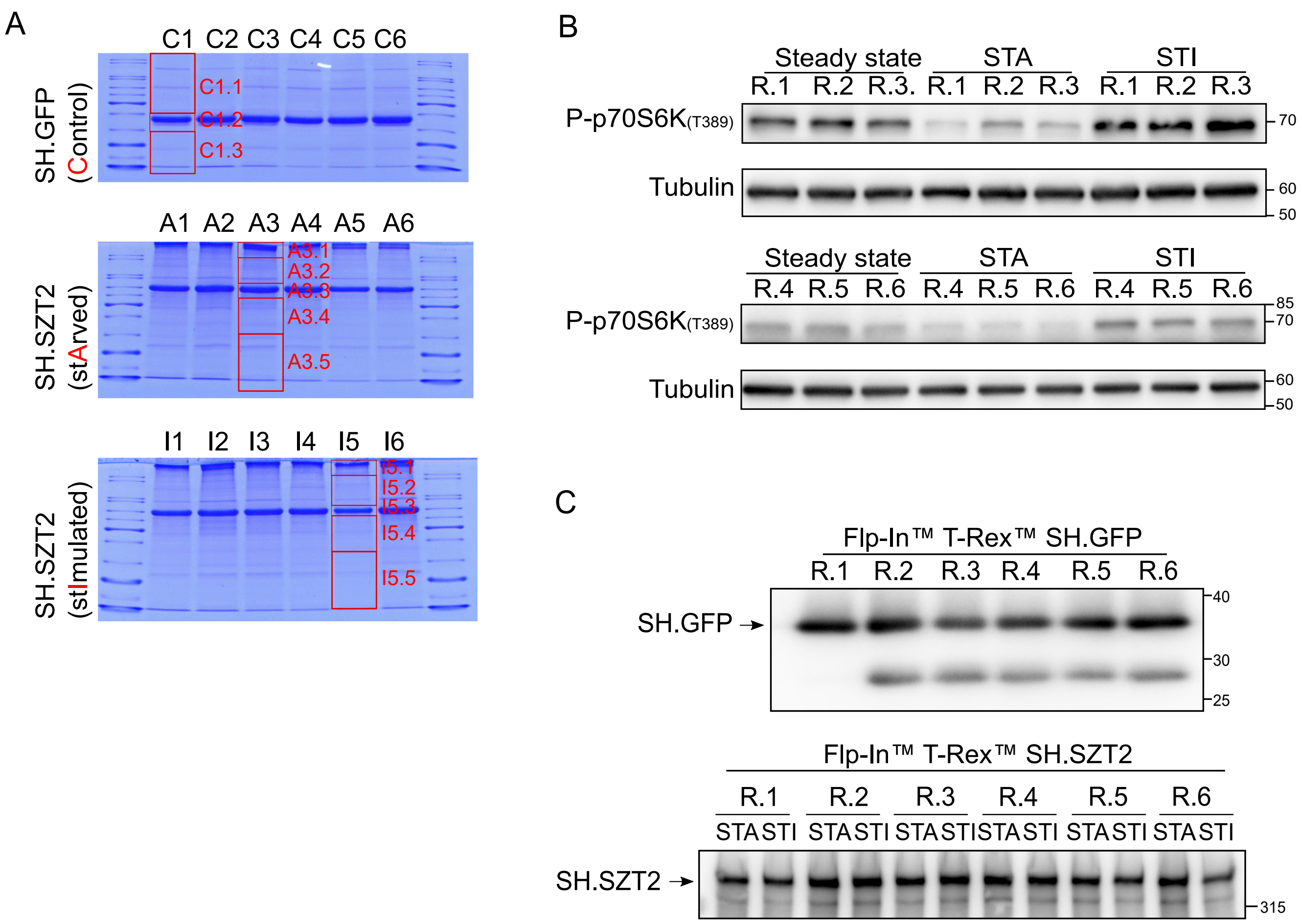
A
C1 C2 C3 C4 C5 C6
C1.1
C1.2
C1.3
SH.GFP (Control)
A1 A2 A3 A4 A5 A6
A3.1
A3.2
A3.3
A3.4
A3.5
SH.SZT2 (stArved)
I1 I2 I3 I4 I5 I6
I5.1
I5.2
I5.3
I5.4
I5.5
SH.SZT2 (stImulated)
B
Steady state
STA
STI
R.1 R.2 R.3. R.1 R.2 R.3 R.1 R.2 R.3
P-p70S6K(T389)
70
Tubulin
60
50
Steady state
STA
STI
R.4 R.5 R.6 R.4 R.5 R.6 R.4 R.5 R.6
85
70
P-p70S6K(T389)
Tubulin
60
50
C
Flp-In™ T-Rex™ SH.GFP
R.1 R.2 R.3 R.4 R.5 R.6
SH.GFP
40
30
25
Flp-In™ T-Rex™ SH.SZT2
R.1 R.2 R.3 R.4 R.5 R.6
STA STI STA STI STA STI STA STI STA STI STA STI
SH.SZT2
315

Figure S1

GFPvsSZT2 Ratio>5 P<0.01

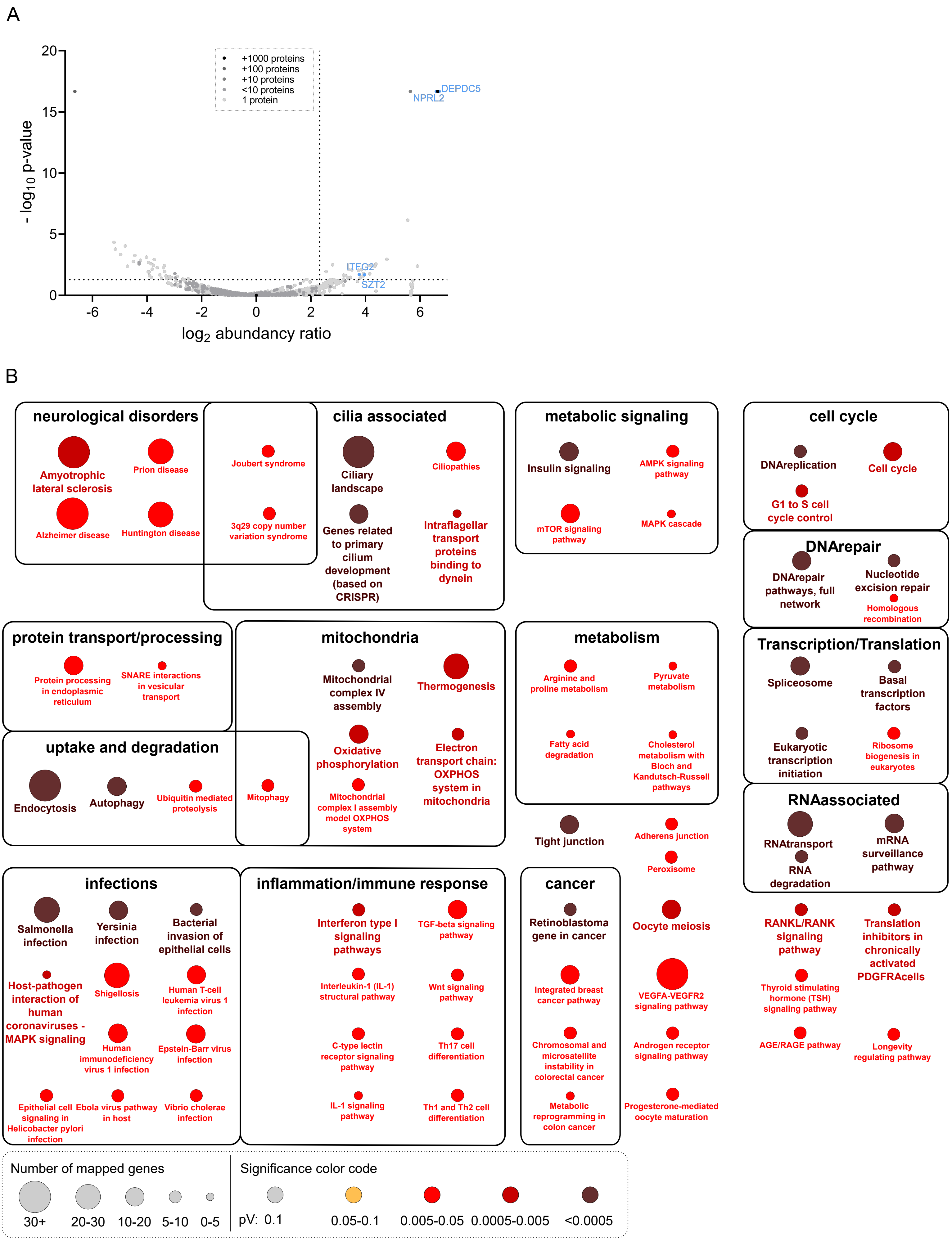


FIGURE S2

A

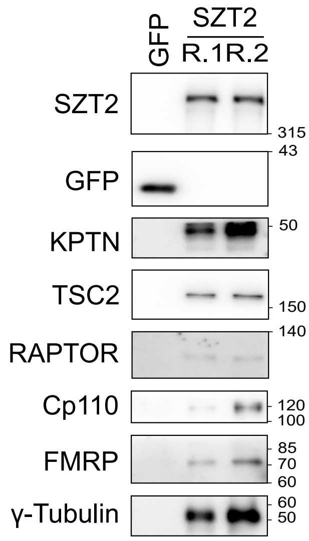


B

| Gene Symbol | UniProt P. access.nr. | Description | STA vs. ctrl | | STI vs. ctrl | | STI vs. STA | |
|---|---|---|---|---|---|---|---|---|
| | | | ratio | p-value | ratio | -p-value | ratio | p- value |
| FMR1 | Q06787 | Synaptic functional regulator FMR1 | 100 | 2.063E-17 | 100 | 2.080E-17 | 0.68 | 0.67 |
| NUFIP1 | Q9UHK0 | Nuclear fragile X mental retardation-interacting protein 1 | 100 | 2.063E-17 | 100 | 2.080E-17 | n.a. | n.a. |
| BNIP1 | Q12981 | Vesicle transport protein SEC20 | 0.01 | 2.063E-17 | 93.62 | 7.18E-07 | 100 | 3.45E-17 |
| TEX264 | Q9Y6I9 | Testis-expressed protein 264 | 100 | 2.063E-17 | 100 | 2.080E-17 | 0.98 | 0.93 |
| PEX14 | O75381 | Peroxisomal membrane protein PEX14 | 100 | 2.063E-17 | 100 | 2.080E-17 | 0.89 | 0.99 |
| USP14 | P54578 | Ubiquitin carboxyl-terminal hydrolase 14 | n.a. | n.a. | 100 | 2.080E-17 | 100 | 3.45E-17 |
| LGALS3 | P17931 | Galectin-3 | 100 | 2.06E-17 | 100 | 2.080E-17 | 1.35 | 0.37 |
| SNAP29 | O95721 | Synaptosomal-associated protein 29 | n.a. | n.a. | 100 | 2.080E-17 | 100 | 3.45E-17 |

FIGURE S3

## Genotyping

A

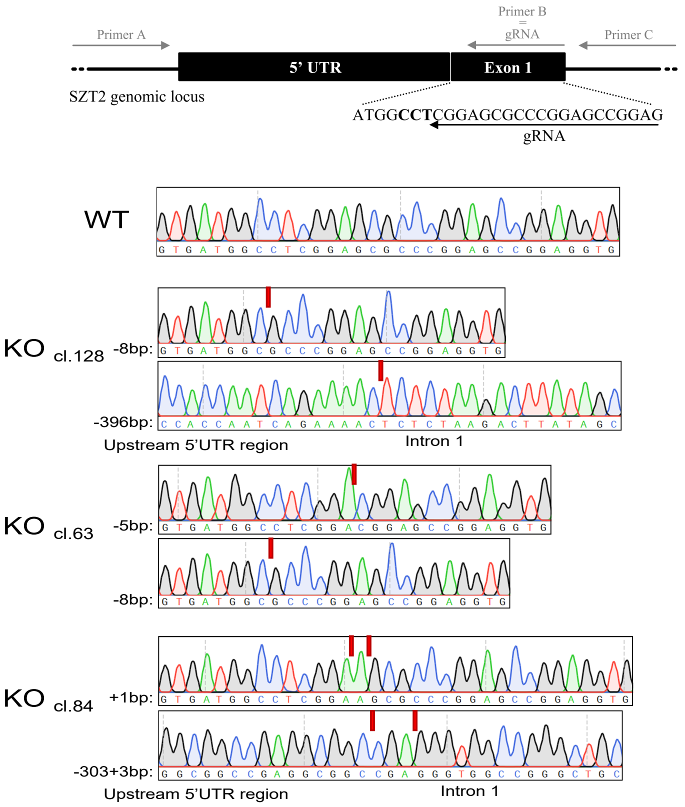


FIGURE S4

# Amino acids and FBS sensing

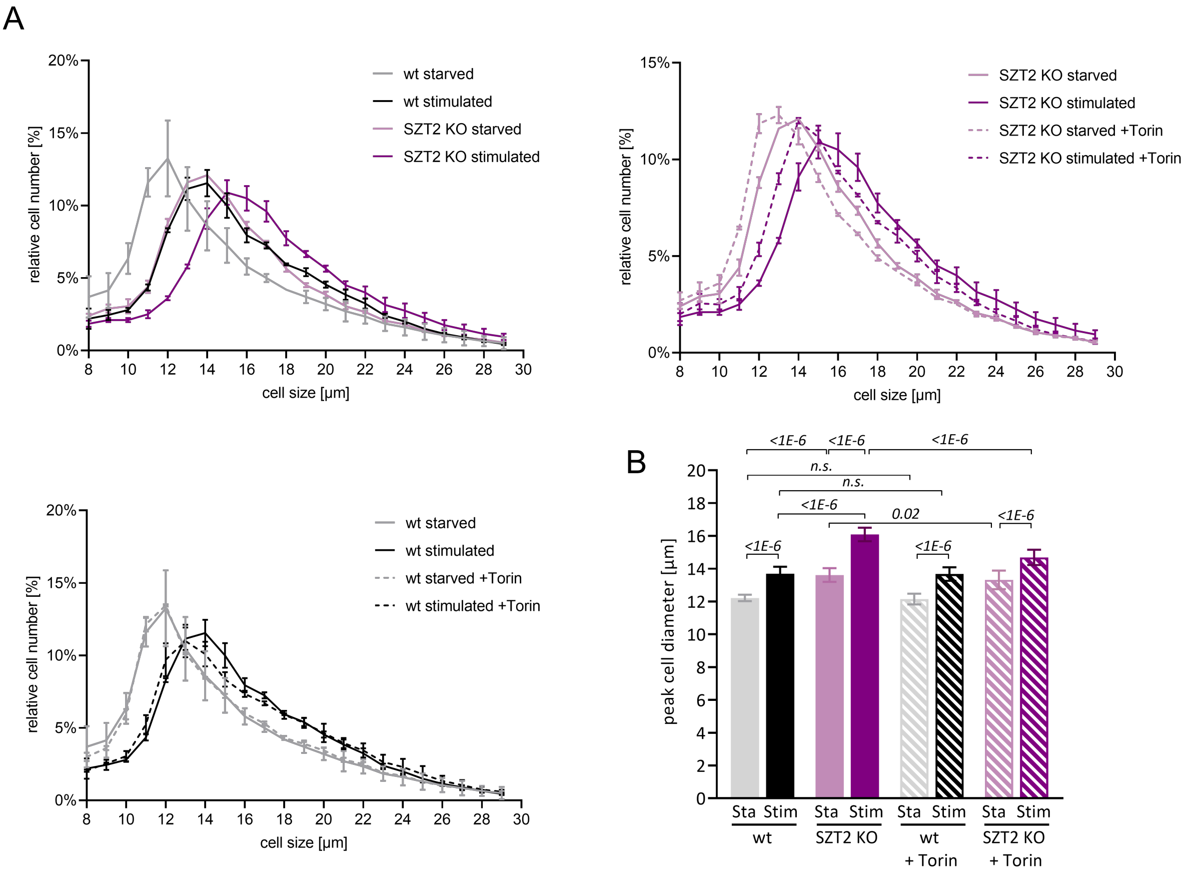


FIGURE S5

A

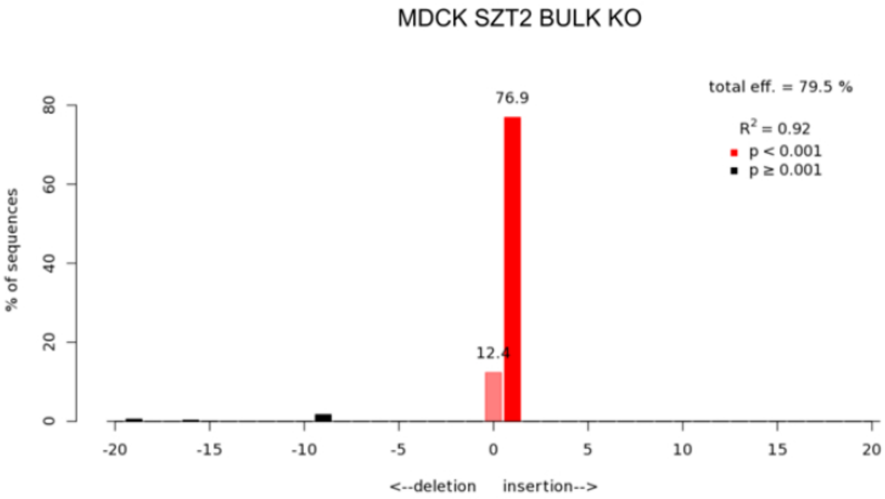


B

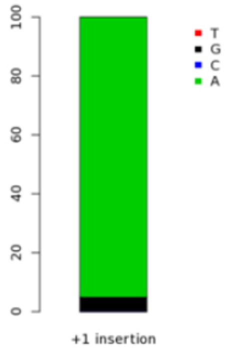


| A | C | G | T |
|---|---|---|---|
| 95.0 | 0.0 | 5.0 | 0.0 |

FIGURE S6
